# Integration analysis of metabolome and transcriptome reveals the effect of exogenous supplementation with mixtures of vitamins ADE, zinc, and selenium on follicular growth and granulosa cells molecular metabolism in donkeys (*Equus asinus*)

**DOI:** 10.3389/fvets.2022.993426

**Published:** 2022-10-28

**Authors:** Yajun Guo, Weisen Zhao, Nan Li, Shizhen Dai, Hao Wu, Zhenlong Wu, Shenming Zeng

**Affiliations:** ^1^National Engineering Laboratory for Animal Breeding, Key Laboratory of Animal Genetics and Breeding of the Ministry of Agriculture, College of Animal Science and Technology, China Agricultural University, Beijing, China; ^2^Department of Clinical Sciences, College of Veterinary Medicine, China Agricultural University, Beijing, China; ^3^State Key Laboratory of Animal Nutrition, College of Animal Science and Technology, China Agricultural University, Beijing, China

**Keywords:** vitamins ADE, Zn, Se, granulosa cells, steroidogenesis pathway, mineral metabolism, metabolome, transcriptome

## Abstract

Vitamins and microelements play essential roles in mammalian ovarian physiology, including follicle development, ovulation, and synthesis and secretion of hormones and growth factors. However, it is nevertheless elusive to what extent exogenous supplementation with mixtures of vitamins ADE, zinc (Zn), and selenium (Se) affects follicular growth and granulosa cells (GCs) molecular function. We herein investigated their effect on follicular growth and GCs physiological function. We showed that follicular growth and ovulation time was accelerated and shortened with the increases of vitamins ADE, Zn, and Se doses by continually monitoring and recording (one estrus cycle of about 21 days) with an ultrasound scanner. Integrated omics analysis showed that there was a sophisticated network relationship, correlation expression, and enrichment pathways of the genes and metabolites highly related to organic acids and their derivatives and lipid-like molecules. Quantitative real-time PCR (qPCR) results showed that vitamin D receptor (*VDR*), transient receptor potential cation channel subfamily m member 6 (*TRPM6*), transient receptor potential cation channel subfamily v member 6 (*TRPV6*), solute carrier family 5 member 1 (*SLC5A1*), arachidonate 5-lipoxygenase (*ALOX5*), steroidogenic acute regulatory protein (*STAR*), prostaglandin-endoperoxide synthase 2 (*PTGS*2), and insulin like growth factor 1 (*IGF-1*) had a strong correlation between the transcriptome data. Combined multi-omics analysis revealed that the protein digestion and absorption, ABC transporters, biosynthesis of amino acids, aminoacyl-tRNA biosynthesis, mineral absorption, alanine, aspartate and glutamate metabolism, glycine, serine and threonine metabolism, arginine biosynthesis, and ovarian steroidogenesis were significantly enriched. We focused on the gene-metabolite interactions in ovarian steroidogenesis, founding that insulin receptor (*INSR*), phospholipase a2 group IVA (*PLA2G4A*), adenylate cyclase 6 (*ADCY6*), cytochrome p450 family 1 subfamily b member 1 (*CYP1B1*), protein kinase camp-activated catalytic subunit beta (*PRKACB*), cytochrome p450 family 17 subfamily a member 1 (*CYP17A1*), and phospholipase a2 group IVF (*PLA2G4F*) were negatively correlated with β-estradiol (E_2_), progesterone (P_4_), and testosterone (T) (*P* < 0.05). while *ALOX5* was a positive correlation with E2, P_4_, and T (*P* < 0.05); cytochrome p450 family 19 subfamily a member 1 (*CYP19A1*) was a negative correlation with cholesterol (*P* < 0.01). In mineral absorption, our findings further demonstrated that there was a positive correlation between solute carrier family 26 member 6 (*SLC26A6*), *SLC5A1*, and solute carrier family 6 member 19 (*SLC6A19*) with Glycine and L-methionine. Solute carrier family 40 member 1 (*SLC40A1*) was a negative correlation with Glycine and L-methionine (*P* < 0.01). *TRPV6* and ATPase Na+/K+ transporting subunit alpha 1 (*ATP1A1*) were positively associated with Glycine (*P* < 0.05); while ATPase Na+/K+ transporting subunit beta 3 (*ATP1B3*) and cytochrome b reductase 1 (*CYBRD1*) were negatively related to L-methionine (*P* < 0.05). These outcomes suggested that the vitamins ADE, Zn, and Se of mixtures play an important role in the synthesis and secretion of steroid hormones and mineral absorption metabolism pathway through effects on the expression of the key genes and metabolites in GCs. Meanwhile, these also are required for physiological function and metabolism of GCs. Collectively, our outcomes shed new light on the underlying mechanisms of their effect on follicular growth and GCs molecular physiological function, helping explore valuable biomarkers.

## Introduction

Donkeys (*Equus asinus*) are one of the most common species that provide labor in agriculture and transportation (1). With the wide application of modern means of transport, less attention is given to the donkey as a means of transport, resulting in a dramatic decline in donkey population sizes in the past three decades. Fortunately, their meat, skin, and milk are attracting increasing attention due to the considerable commercial and economic benefits and medical value (2). Meanwhile, many breeds of donkeys now have small population sizes and the risks of inbreeding are high; therefore, the genetic diversity and reproductive performance of donkeys have declined (3). In recent years, our research group has been committed to studying the regularity of the estrus cycle, dynamic changes in follicular development, control of ovulation time, and timed artificial insemination, which may provide a theoretical basis and technical support for improvement in reproduction (4, 5). There are many factors affecting the fecundity of jennies, among which nutrient level plays a very important role in the estrus cycle, follicular development, and ovulation. However, vitamin and mineral deficiencies may decrease production and cause clinical consequences, such as equine motor neuron disease, oxidative damage, or decreased immune response (6). Studies have reported that vitamins ADE, Zn, and Se directly or indirectly affect mammalian reproduction through their involvement in the synthesis and secretion of hormones (7, 8). Se and vitamin E deficiencies may threaten the health status of production donkeys, especially the health of a newborn foal, growing donkey, and lactating jenny (9). Previous reports have revealed that vitamin A is a fat-soluble vitamin, which regulates the growth and development of animals, cell proliferation and differentiation, and the maintenance of tissue function by affecting steroid synthesis (10). Vitamin D is a steroid hormone, synthesized mainly by the skin on exposure to ultraviolet light, with <10–20% coming from diet (11). It is converted to 25-hydroxyvitamin D by hepatic 25-hydroxylase; then circulating 25-hydroxyvitamin D gets converted by renal 1a-hydroxylase to the active form 1,25-dihydroxyvitamin D3 (12, 13). Studies have reported that several GCs express the cytochrome p450 family 27 subfamily b member 1 (*CYP27B1*) enzyme, *VDR*, and Vitamin D-Binding Protein (VDBP), and *in vitro* treatment with functional Vitamin D3 (VD3) enhanced the survival of primate preantral and antral follicles and was associated with significantly larger antral follicles (14, 15). In summarizing, the above studies obviously demonstrate that vitamins and micronutrients play an essential role in the reproductive physiology of the ovary. Additionally, Vitamin E is also a fat-soluble antioxidant and a cell membrane antioxidant that enhances immune responses in a variety of animals (16). One study suggested that it prevents lipid peroxidation and protects the antioxidant system in the mouse ovary (17). Otherwise, vitamin E, as a cofactor of glutathione peroxidase, plays an important role in the removal of reactive oxygen species (ROS) from the ovary. Another study suggested that supplementation with vitamin E can increase the level of the anti-mullerian hormone (AMH), antral follicle count, and mean ovarian volume in women with occult premature ovarian insufficiency (POI) (17, 18). Studies have shown that Se accumulates in the GCs of healthy and large follicles but is not found in the small and atretic follicles in the ovary (19–21), which suggest that selenium is associated with large follicle growth and development. Zn is an essential microelement that plays many important roles in maintaining homeostasis. It is crucial for the regulation of cell growth, hormone release, immunological response, and reproduction (22). Because the cells of the reproductive system differentiate and proliferate considerably, processes that include spermatogenesis, ovulation, fertilization, normal pregnancy, embryo development, and parturition are Zn-dependent, and Zn is essential for the proper functioning of the reproductive system (23–25). In summary, these findings suggest that vitamins ADE, Zn, and Se mixtures are essential for reproductive performance. However, how it affects follicular development, GCs proliferation, differentiation, and ovulation in jennies remain poorly understood. To determine whether vitamins ADE, Zn, and Se act as moderators of follicular growth and GCs proliferation. Combined metabolomics and transcriptomics were performed to construe the network regulatory relationships between metabolites and genes in the mineral absorption and ovarian steroidogenesis pathways. Our outcomes highlight the association between vitamins ADE, Zn, and Se and follicular and GCs development, which may provide key insights into reproductive biology and fertility.

## Materials and methods

A total of 40 jennies were fed and treated humanely in accordance with the Ethical Guidelines of Agricultural Animals (no. 11002009000012, production license number: SCXK: 2012-0766, China). The used protocols were reviewed by the Institutional Animal Care Committee of China Agricultural University.

### Feeding of animals

This study was conducted at the Zhangjiakou Sangyang Herding Co., Heibei Province, China (latitude 39° 53′-40° 22′ N and longitude 113° 54′-114° 48′ E, at approximately 1100 m above sea level) from 2021 to 2022. A herd of 40 jennies (Yangyuan donkey, aged: 3~4 years, average body weight: 292.946 ± 33.543 kg) was used for this experiment. Ensuring similar weights, ages, and sizes across groups, the jennies were randomly divided into four groups of 10 jennies each: A, B, C, and D. Each group was housed in a spacious and ventilated stall, approximately 30 m in length and 20 m in width. All the jennies were fed according to the nutrition formulation (Supplementary Table 1) twice daily, which was meant to maintain normal demand and healthy body condition. Additionally, based on the above feeding regime, groups A, B, C, and D were fed different concentrations-150, 100, 75, and 50%, respectively-of vitamins ADE, Zn, and Se. Its concentrations were set according to the National Research Council's standard for mares, as shown in Supplementary Table 2.

### Sample collection

The mixture was instilled into the mouth of each jenny at a dose of 5 mL each using a syringe without a needle once daily for 2 months (Supplementary Figure 1A). After the 2 months of feeding, each group (*n* = 10) was observed for 3 consecutive estrous cycle (one estrus cycle of about 21 days) using ultrasound scanner with a 6.5 MHz linear array probe (KX5600, Kaixin Electronic Instrument Co., Ltd, Xuzhou, China) to measure follicle diameter, dynamic changes in follicle growth, ovulation time, double dominant follicles, and ovulation number. The detailed operation and the ultrasound images of the follicles was shown in the Supplementary Information (SI) and Supplementary Figures 1B,C.

The measurement imaging system of the transvaginal ultrasound-guided follicle aspiration device (Easi-Scan Micro-Convex, BCF Technology Ltd, Scotland, UK; Probe parameter: 15 mm radius, frequency range 5–8 MHz, 80 element crystal array, 10 digital channels, 90° curved) was utilized to obtain dominant follicles (diameter 30-40mm) with ultrasound guidance. Each jenny's follicles were collected across three estrous cycles (two follicles can be collected per jenny in one estrous cycle). The specific operation method was detailed in Supplementary Information (SI) Appendix. Next, under a somatic microscope, the cumulus-oocyte complexes (COCs) of follicular fluid (FF) were then eliminated before being filtered through a cell sieve (70μm). From each group, GCs (*n* = 4, six follicles per head) and FF (*n* = 6, six follicles per head) were obtained. In brief, the FF was obtained by centrifugation at 500 g for 5 min, the supernatant solution was collected and frozen at −80°C for non-targeted metabolomics analysis. Collected GCs were washed with PBS and re-suspended three times by centrifugation at 300 g for 5 min to collect the precipitation. It was stored at −80°C for RNA-Seq and qPCR analysis.

### Total RNA extraction and transcriptome sequencing

Total RNA of the GCs was extracted using Trizol reagent (Takara, Dalian, China) according to the operation specifications. The libraries for transcriptome sequencing were constructed using the Nebnext Ultra Directional RNA Library Prep Kit for Illumina (NEB, CA). Transcriptome sequencing was performed using an Illumina Novaseq 6000 sequencer. The reference genome index of Donkey (*Equus asinus*) was built using the program Bowti2 (version 2.2.6) with default parameters. Gene expression abundance quantification was performed using the programs HTSeq (version 0.9.1) and DESeq (version 1.30.0) with default parameters. Expressed genes with |log2 (fold change)| ≥ 1 and adjusted *P*-values of *P* < 0.05 were considered differentially expressed genes (DEGs). Correlation analysis using Pearson's correlation coefficient was employed to represent the correlation of gene expression levels between samples. When 0.8 < R < 1, the expression pattern between samples indicated a higher similarity. GO enrichment analysis (http://geneontology.org/) was used to calculate the gene list and the number of genes in each term, and the standard of significant enrichment was *P* < 0.05. Kyoto Encyclopedia of Genes and Genomes (KEGG) enrichment analysis (https://www.KEGG.Jp/) was used to calculate the gene list and gene number of each pathway, and *P* < 0.05 was regarded as the standard of significant enrichment.

### Metabolite extraction and metabolomic profiling

For liquid chromatography-mass spectrometry (LC-MS) analysis, the follicular fluids were redissolved in acetonitrile/water (1:1, v/v) solvent. Analyses were performed using a UHPLC (1290 Infinity LC, Agilent Technologies) coupled to a quadrupole time-of-flight (AB Sciex TripleTOF 6600). In ESI positive and ESI negative modes, the instrument was set to acquire data over the m/z range 25–1000 Da, and the accumulation time for the production scan was set at 0.05 s/spectra. Metabolomic profiling was performed using a software analyst (AB Sciex, Darmstadt, Germany). After being normalized to the total peak intensity, the processed data were analyzed using the R software package, in which it was subjected to multivariate data analysis, particularly Pareto-scaled principal component analysis (PCA) and orthogonal partial least-squares discriminant analysis (OPLS-DA). The seven-fold cross-validation and response permutation testing was used to evaluate the robustness of the model. The variable importance in the projection (VIP) value of each variable in the OPLS-DA model was calculated to indicate its contribution to the classification. Metabolite identification was performed based on the KEGG database (https://www.genome.jp/kegg/), the Human Metabolome Database (https://hmdb.ca/metabolites), and the Lipid Maps Database (https://www.lipidmaps.org/). Differentially accumulated metabolites (DAMs) were evaluated using the VIP scores *via* the PLS-DA model with thresholds of VIP ≥ 1, fold change ≥ 1.5 or ≤ 0.667, and *P* < 0.05. The metabolites were subsequently analyzed using the above standard filters. The subsequent analysis was only targeted at this part of significant differential metabolites.

### Combined metabolome and transcriptome analysis

Firstly, the relevant information on the metabolites and transcripts was obtained in the KEGG database (*P* < 0.05; https://www.kegg.jp/dbget-bin/www_bfind?compound). Next, we showed the differentially expressed metabolites and their corresponding differential transcripts using interactive network analysis (Cytoscape, https://cytoscape.org/download.html). Finally, genes and metabolites of the key signaling pathway were analyzed using the spearman algorithm for correlation analysis (when −1 < R < 0, the two are negatively correlated; when 0 < R < 1, the two are positively correlated).

### qPCR analysis

Quantitative real-time PCR (qPCR) was performed with the real-time PCR system (Bio-Rad, CFX96Touch, USA) using the HiScript^®^ III-RT SuperMix and Taq Pro Universal SYBR qPCR Master Mix (Nanjing, Vazyme, China) for first-strand cDNA synthesis and preparation of PCR master mix according to the manufacturer's protocols. Specific oligonucleotide primers were synthesized for amplification of targeted genes by Sangon Biotech (Shanghai, China; Supplementary Table 3). The 2^−ΔΔCT^ method was used to determine the relative expression of mRNA using *GAPDH* as control.

### Statistical analysis

The data were subjected to a one-way analysis of variance using SPSS 22.0 (SPSS Inc., Chicago, IL) software. The data from three independent trials are presented as mean ± standard deviation (SD). Dunnett's test was used *post hoc* to determine the differences between the different groups; the differences were considered significant at *P* < 0.05. GraphPad Prism 8.0 software (GraphPad, California, USA) was used for the graphics. The R ggplot2 package (https://www.rproject.org) was used to analyze the DEGs. Metabolites with VIP value > 1 were further subjected to the student's *t*-test at the univariate level to measure the significance of each metabolite; *P* < 0.05 was considered significant.

## Results

### Effects of vitamins ADE, Zn, and Se on follicular growth in jennies

**Figure 1:**
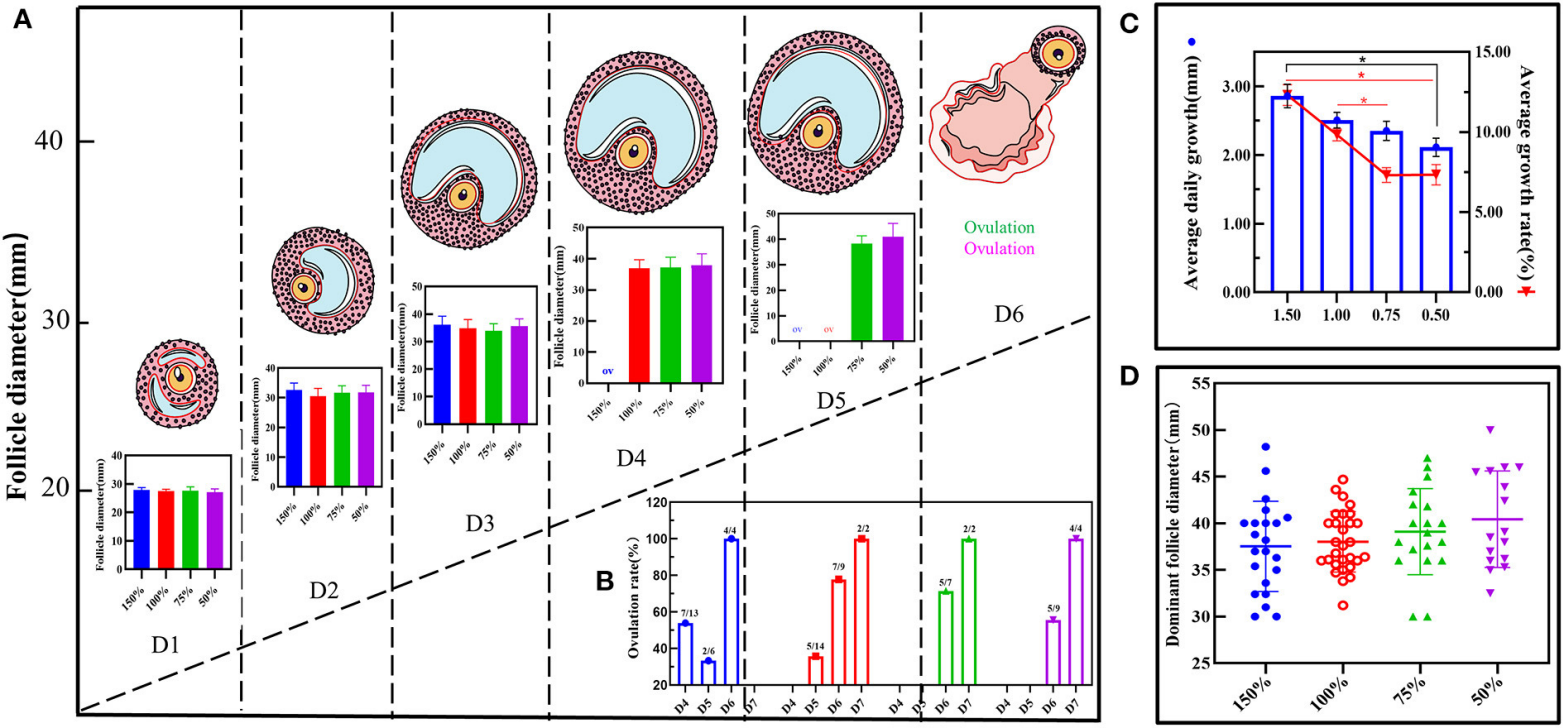
Effect of vitamin ADE, Zn, and Se mixture on follicular growth. **(A)** Dynamic changes in follicular growth were detected using the B-mode ultrasonographic examination. When follicle diameters (Φ = 26–28 mm), it was recorded on the first day; this was indicated as follicular ovulation (Φ = 0 mm). The schematic diagram represents the dynamic visualization of follicular growth until ovulation. The bar graph represents the visualization of the change in the diameter of the growing follicle in different concentration groups. **(B)** The change in ovulation rate in different concentration groups. The number of days spent from follicle diameter (Φ = 26–28 mm) to follicle ovulation (Φ = 0 mm) was calculated for biological statistics for each donkey in each group. **(C)** The change in average daily growth and average growth rate of follicles in different concentration groups. **(D)** The effect of different concentration groups on the dispersion and homogeneity of the diameter of the dominant follicle. To reflect the distribution of the dominant follicles, the number and diameter of the dominant follicles in each group were counted. The *P*-values were determined by two-way ANOVA with Sidak's multiple comparisons test. Data are presented as the mean ± SD of three independent experiments. *P < 0.05, **P < 0.01, ns, not significant.

B-mode ultrasonographic examination was used to obtain follicular growth dynamic changes with follicular diameters from 26–28 mm to preovulation follicles (35–45 mm) (Supplementary Figure 1B). However, there was no significant difference in the diameters among the four groups from day one to the day before ovulation (*P* > 0.05; Figure 1A). In addition, when follicular diameters were from 26–28 mm to 35–45 mm, ovulation time in group D reached 4.90 ± 0.90 days, which was significantly lower than that of the three groups (*P* < 0.05; Figure 1B). With increasing concentration, the doses in the follicular average daily growth and the follicular average growth rate in group D showed a positive correlation, which was significantly different from those of the other three groups (*P* < 0.05; Figure 1C). The dominant follicular diameters reached 37.52 ± 4.73 mm in group D, while they reached 38.02 ± 3.19 mm, 39.11 ± 4.50 mm, and 40.44 ± 5.01 mm in groups C, B, and A, respectively. Notably, the diameter corresponding to the number of dominant follicles in different estrous cycles were relatively neat in group C (Figure 1D). However, by comparison, the dominant follicular bilateral rate was higher in group B and the bilateral ovulation rate was better in group C (Supplementary Table 4). These data showed that vitamins ADE, Se, and Zn may promote follicular development, shorten ovulation time, and increase ovulation rate.

### DEGs of GCs in response to vitamins ADE, Zn, and Se stress

**Figure 2:**
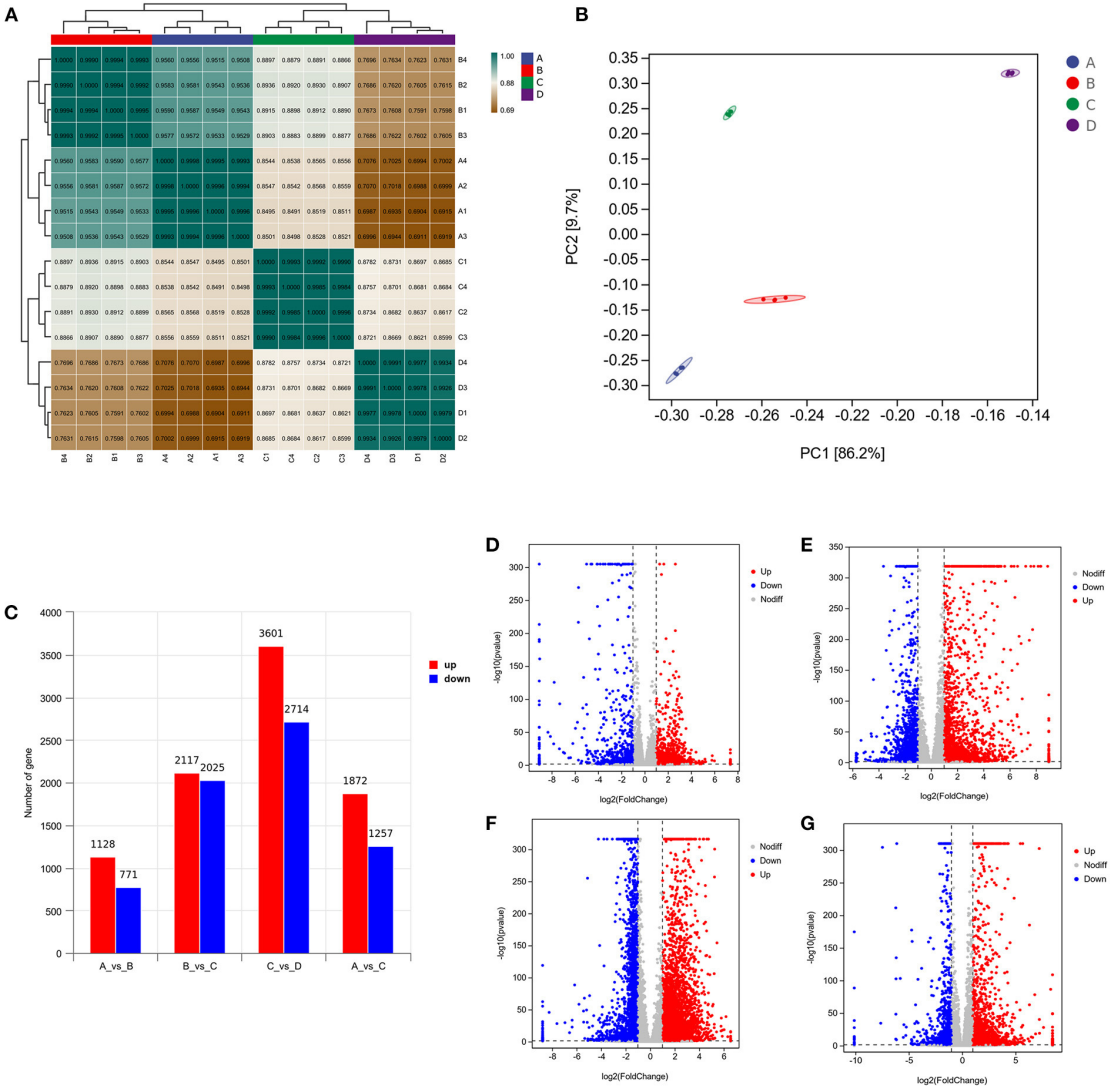
Analysis of DEGs in the four groups. **(A)** Correlation analysis between the four groups. The left and upper sides are sample clustering, the right and lower sides of the figure are sample names, and squares of different colors represent the degree of correlation between the two samples. **(B)** Principal component analysis of samples from four groups. **(C)** Number of DEGs in between the four groups. **(D–G)** Volcano plot showing the DEGs in A vs. B, B vs. C, C vs. D, and A vs. C.

**Figure 3:**
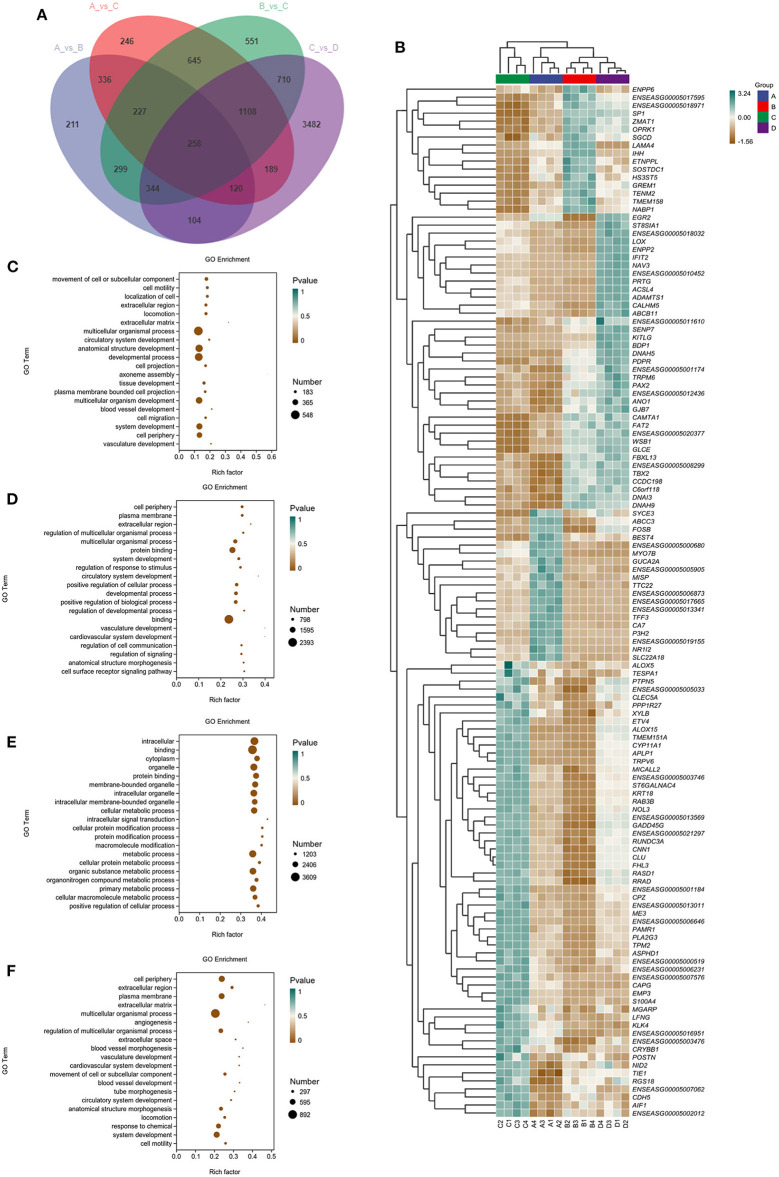
Venn diagram, clustering analysis, and GO enrichment analysis of DEGs within the four groups. **(A)** The Venn diagram has common and unusual patterns in different comparison groups; is the accumulation patterns of shared 258 DEGs in distinct groups. **(B)** In Hierarchical Clustering, the relative levels of 258 DEGs are depicted. **(C–F)** GO enrichment analysis of DEGs in A vs. B, B vs. C, C vs. D, and A vs. C. Rich Factor is the x-coordinate, GO Terms is the ordinate, the size of the point in the image reflects the number of genes annotated in the corresponding term, and the color depth indicates the significance level.

**Figure 4:**
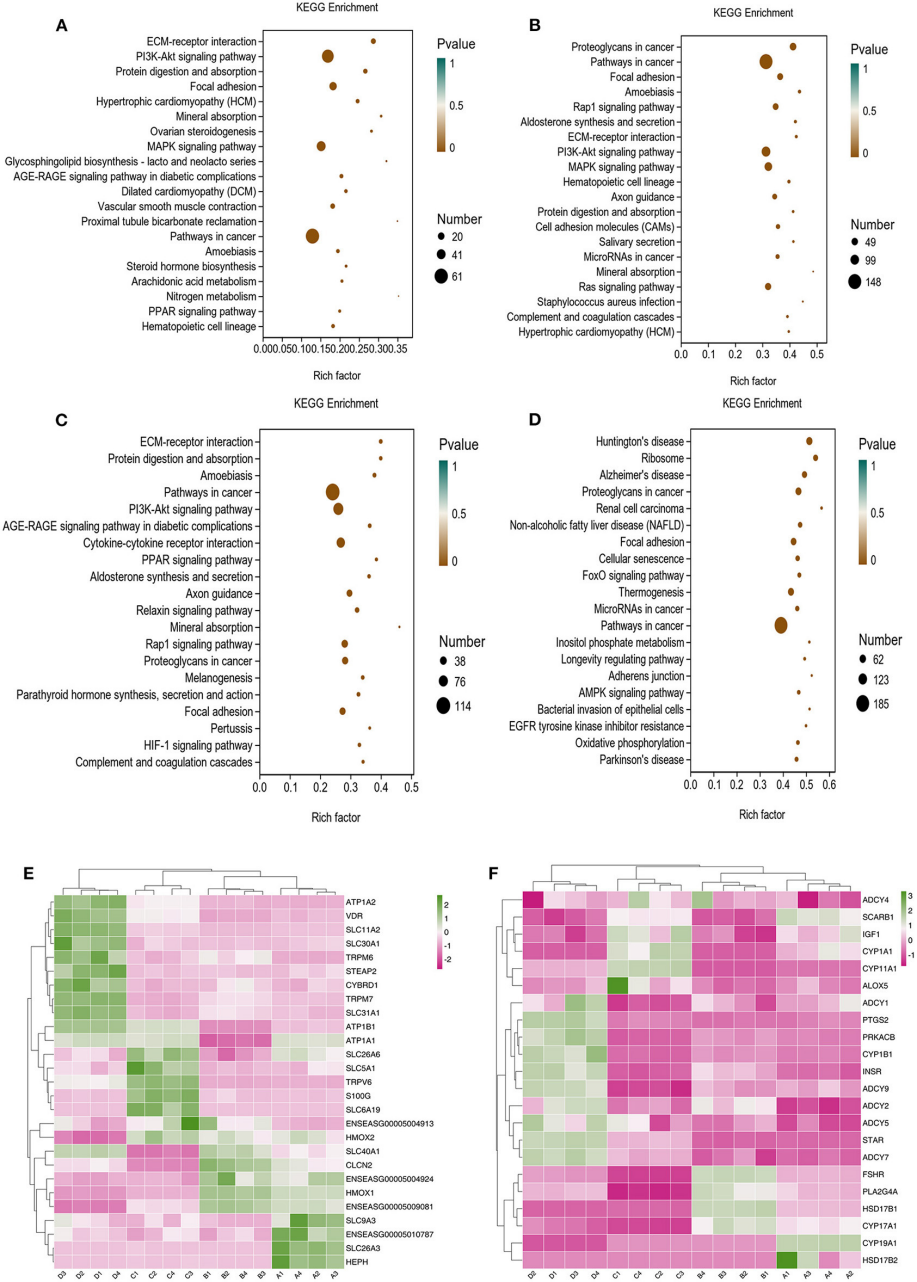
KO analysis of DEGs in Donkey granule cells in the four groups. **(A–D)** Top 20 KEGG enrichment pathways of DEGs. The ordinate is the pathway, and the abscissa is the enrichment factor. Shades of color indicate q-values. Pathway enrichment analysis of DEGs in A vs. B **(A)**, B vs. C **(B)**; C: A vs. C **(C)**, and C vs. D **(D)**. Cluster analysis expression level of revealed mineral absorption **(E)** and ovarian steroidogenesis pathway **(F)** enriched genes.

**Figure 5:**
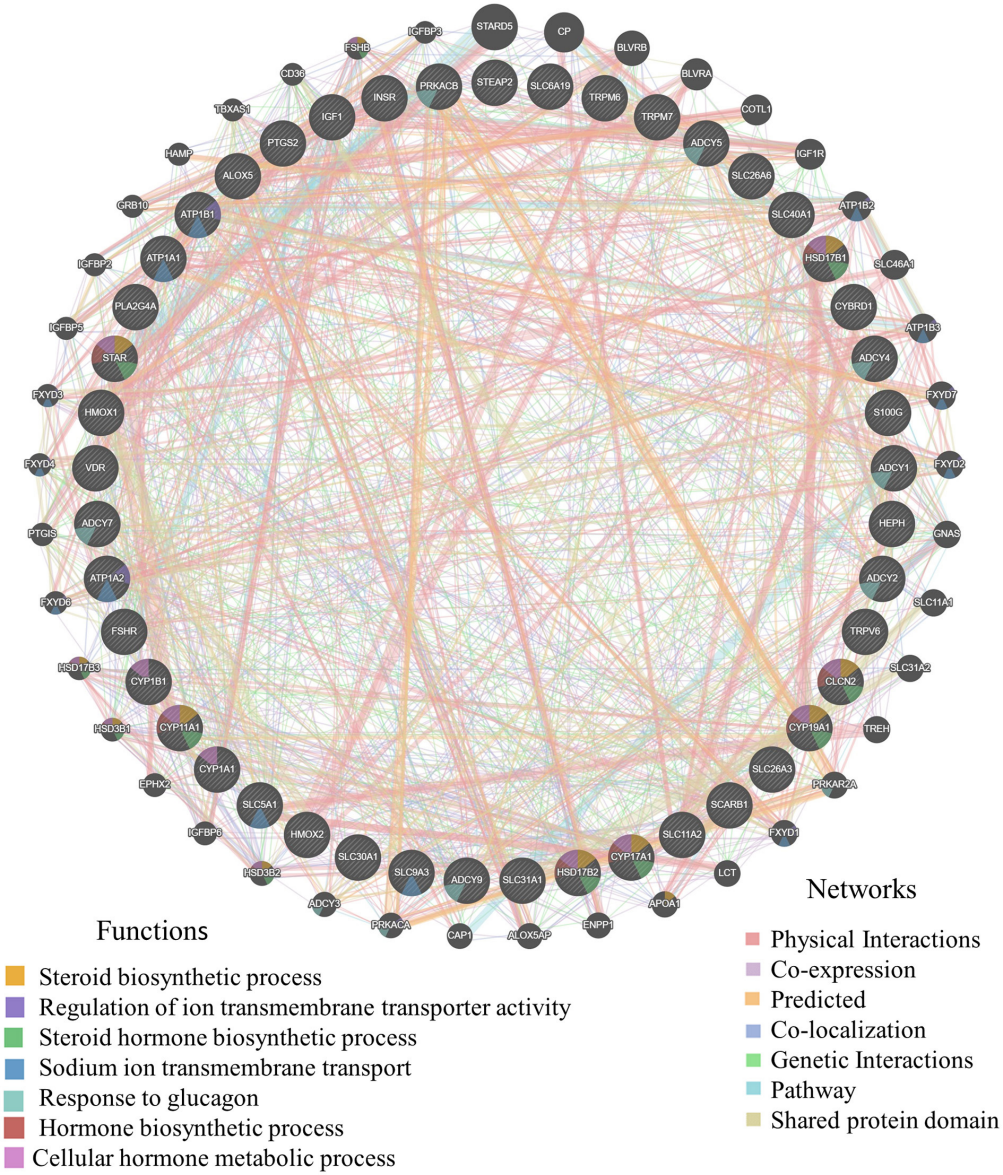
The gene interaction of mineral absorption and ovarian steroidogenesis. Germania protein-protein interaction network was generated with the aforementioned DEGs using online analysis (http://genemania.org/). The thicker the line between genes, the higher the correlation of genes; the higher the number of lines between the gene and other genes, the more important the gene.

To provide a system-wide overview of vitamin and microelements changes in gene expression during follicular development, transcriptomic profiles were investigated using RNA-Seq. After quality filtering, a total of 33,125,638–42,640,498 clean paired reads were obtained, with 88.38–91.90% of clean reads mapped to the donkey reference genome (Supplementary Table 5). Correlation analysis and cluster analysis were performed to understand the relationships among the different samples. Almost all the biological duplicates were also clustered, confirming the relevance of the samples (Figures 2A,B). All the DEGs of four groups were classified using hierarchical clustering and gene-expression trends were divided into nine clusters (Supplementary Figure 2). A total of 1,899 DEGs (1,128 upregulated and 771 downregulated) were identified in group A vs. B; in groups B vs. C, 2,117 upregulated and 2,025 downregulated DEGs were identified; in group C vs. D, 3,601 upregulated and 2,714 downregulated DEGs were identified; and in group A vs. C, 1,872 upregulated and 1,257 downregulated DEGs were identified (Figure 2C). Volcano plots were used to illustrate the distribution of the DEGs between different groups (Figures 2D–G). In addition, to know the co-expressed genes of the four groups, Venn diagram analyses were performed. 258 differential genes were co-expressed in groups A vs. B, B vs. C, C vs. D, and A vs. C; these DEGs were demonstrated in the different groups using hierarchical clustering (Figures 3A,B). To further understand the functions of these DEGs, GO and KEGG analyses were performed. A total of 9,352 GO terms and 295 pathways were significantly enriched in all the groups. The top 20 significantly enriched GO terms are shown in Figures 3C–F. Interestingly, within biological processes, the top three significantly enriched GO terms were the movement of a cell or subcellular component (GO:0006928), cell motility (GO:0048870), and cell localization (GO:0051674). However, in cellular components, the top three significantly enriched GO terms were extracellular region (GO: 0005576), extracellular matrix (GO: 0031012), and cell projection (GO: 0042995). In the molecular functions, the top three significantly enriched GO terms were protein binding (GO: 0005515), signaling receptor binding (GO: 0005102), and cell adhesion molecule binding (GO: 0050839). The top 20 significantly enriched KEGG pathways are shown in Figures 4A–D. Significantly enriched pathways were mainly associated with the pattern of signaling molecules and interaction, signal transduction, digestive system, cellular community-eukaryotes, endocrine system, endocrine, and metabolic diseases, lipid metabolism, immune system processing in KEGG level-2. The top ten significantly enriched KEGG pathways were ECM-receptor interaction, PI3K-Akt signaling pathway, protein digestion and absorption, focal adhesion, mineral absorption, ovarian steroidogenesis, and MAPK signaling pathway, steroid hormone biosynthesis, bile secretion, arachidonic acid metabolism, and PPAR signaling pathway. Of note, we focused on the signaling pathways of mineral absorption and ovarian steroidogenesis and showed differential gene expression using clustering analysis (Figures 4E,F). Next, potential interactions of the abovementioned genes were analyzed using the genemania online platform (Figure 5). Based on the DEGs pathway enrichment results, qPCR of the DEGs (*VDR, TRPM6, TRPV6, SLC5A1, ALOX5, STAR, PTGS2*, and *IGF-1*) were related to the regulation of hormones, reproductive process, and mineral absorption. The results showed a strong correlation between the RNA-seq and qPCR data indicating the reliability of the RNA-seq data (Supplementary Figures 3A–H). Together, these data suggested that vitamins ADE, Zn, and Se have important effects on gene expression in the signaling pathways related to GCs development.

### DAMs of GCs under vitamins ADE, Zn, and Se stress

**Figure 6:**
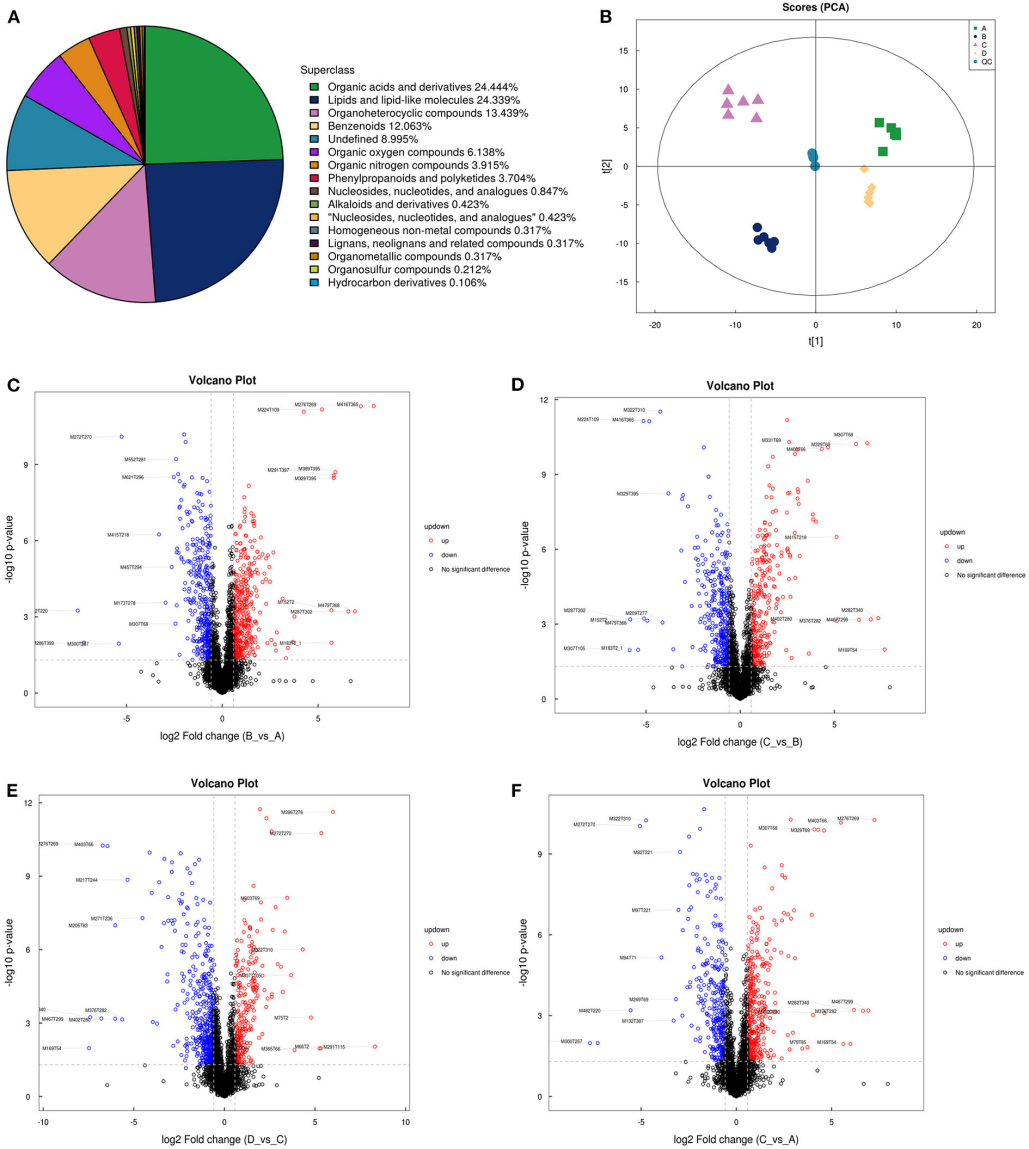
The proportion of identified, principal component analysis, and volcano plots of metabolites. **(A)** The different color blocks in the figure represent different chemical classification items, and the percentage represents the percentage of the number of metabolites in this chemical classification item in all the identified metabolites. **(B)** Principal component analysis in the four groups. Points with the same color represent biological repeats within the groups and the distribution of points reflects the differences between the groups and within the groups. **(C–F**) Volcano plots show patterns between different concentration groups. Each dot represents one metabolite. The red dots represent the significantly upregulated metabolites and the blue dots represent the significantly downregulated metabolites. The black dots represent non-significantly different metabolites.

**Figure 7:**
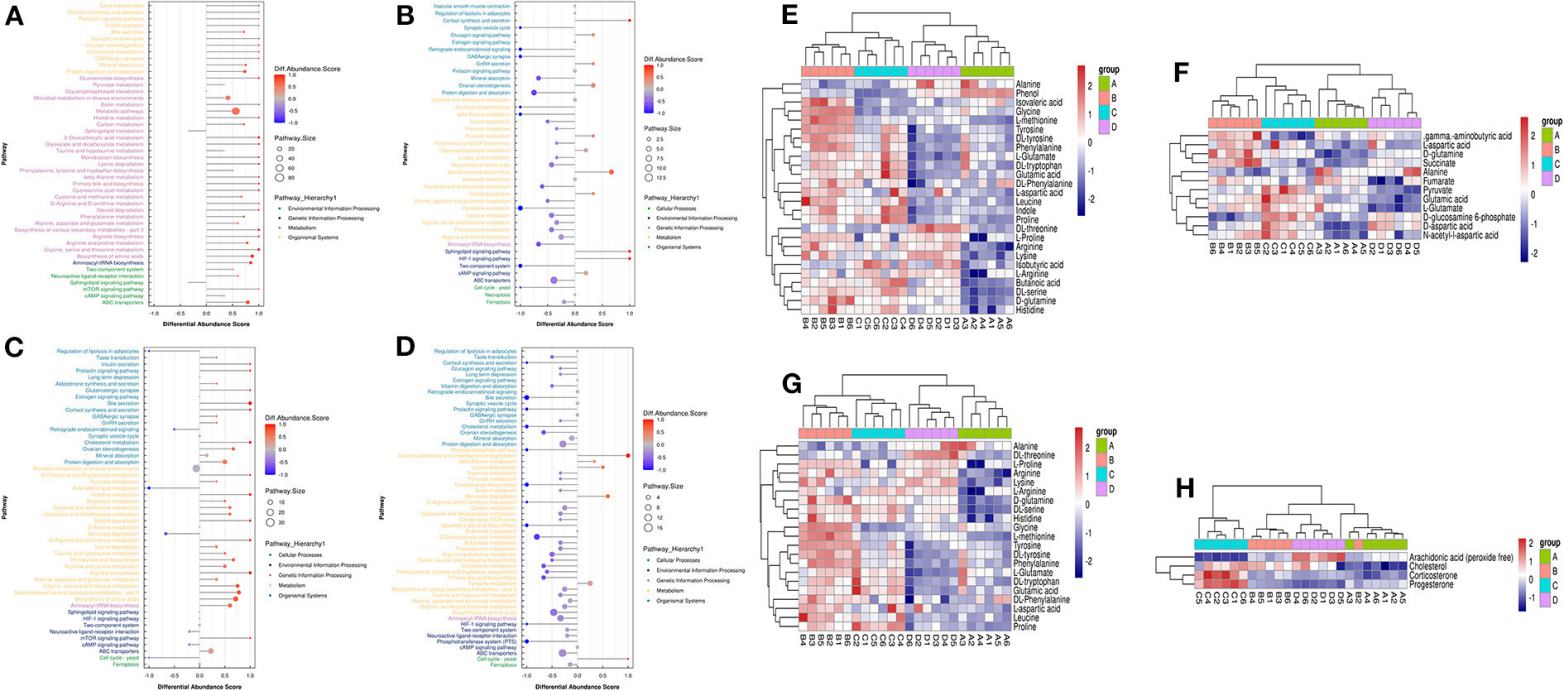
The plot of abundance scores and clustering analysis of DAMs for enrichment pathways. **(A)** Plot of differential abundance scores in A vs. B. **(B)** Plot of differential abundance scores in B vs. C. **(C)** Plot of differential abundance scores in C vs. D. **(D)** Plot of differential abundance scores in A vs. C. **(E–H)** Relative levels of differential metabolites of Protein digestion and absorption, Alanine, Aspartate and Glutamate metabolism, Aminoacyl-tRNA biosynthesis, and Aldosterone synthesis and secretion.

**Figure 8:**
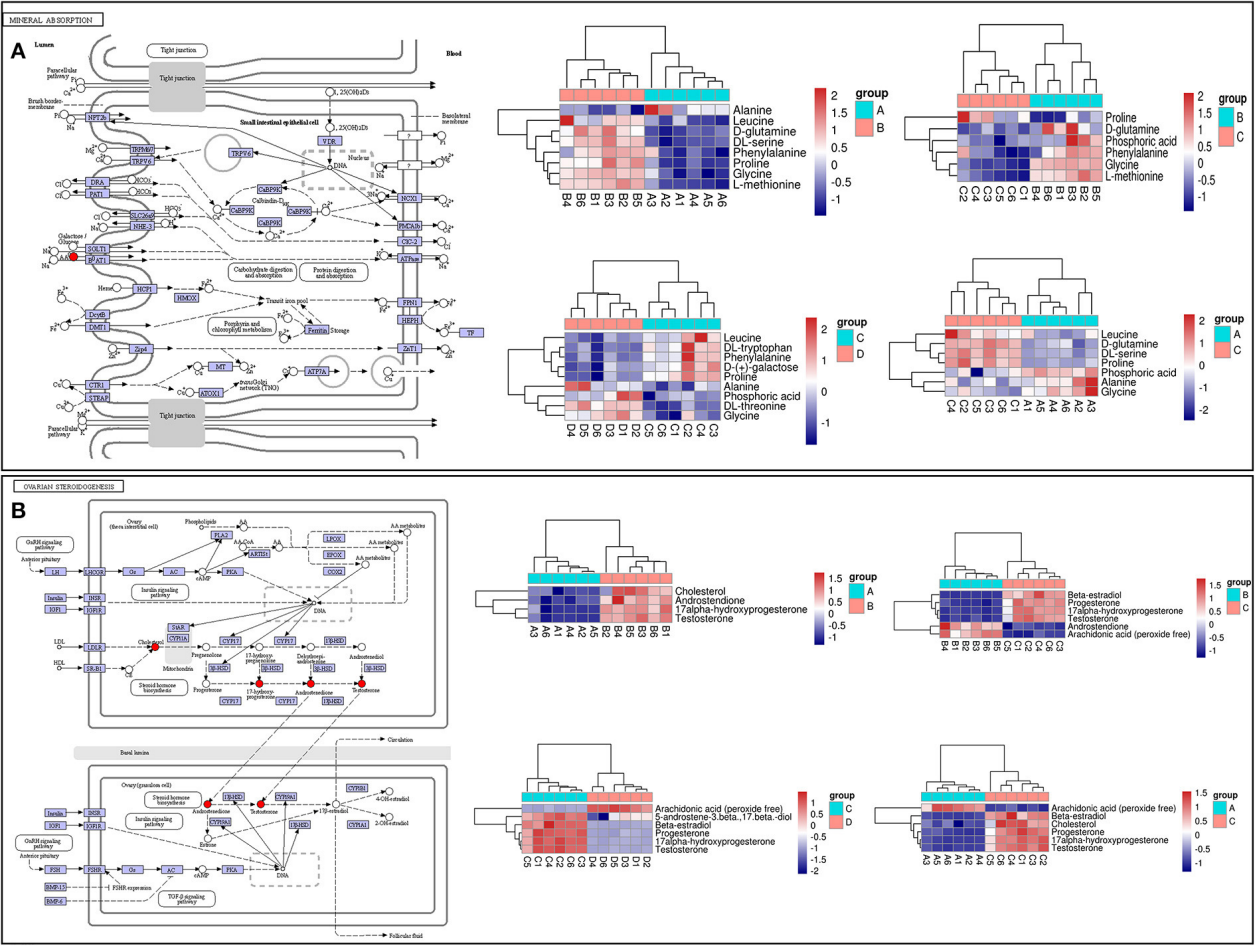
KEGG pathway map and hierarchical clustering heat map of DAMs. **(A)** KEGG map (left) and hierarchical clustering heat map (right) of the expression levels of differential metabolites of mineral absorption. **(B)** KEGG map (left) and hierarchical clustering heat map of the expression levels of differential metabolites of ovarian steroidogenesis.

LC-MS/MS metabolomic profiling was used to investigate the overview of GCs metabolic alterations in response to vitamins ADE, Zn, and Se stress. A total of 945 metabolites (metabolites identified with positive (528) and negative (417) ions) were annotated after a comparison with the self-constructed database. Based on their chemical classification and proportion of the number of various metabolites, the top four were organic acids and their derivatives (24.44%), lipids and lipid-like molecules (24.34%), organic heterocyclic compounds (13.44%), and benzenoids (12.06%; Figure 6A). PCA revealed a clear separation tendency among the A, B, C, and D groups (Figure 6B; Table 1). For further investigation, we integrated the metabolomics data in positive and negative modes. PLS-DA and OPLS-DA contributed to the formation of a multivariate statistical analysis method that also included supervised pattern recognition, and the model indicated a clear distinction between any two groups (Table 1). These multivariate statistical studies revealed that the model showed good validation and accuracy, suggesting that it could be used for further research. Based on univariate analysis, the differential accumulation metabolites (DAMs) in the ESI+ and ESI- models were analyzed on a volcano map; there were 232, 207, 210, and 202 DAMs for groups A vs. B, B vs. C, C vs. D, and A vs. C, respectively (Figures 6C–F). Among them, significantly different metabolites (VIP > 7) associated with lipids and lipid-like molecules, organic acids and derivatives, and organic nitrogen compounds were identified in A vs. C (Supplementary Table 6). Further to showing the expression patterns of metabolites in different samples more comprehensively, by hierarchical cluster analysis, the metabolites with a significant difference are shown in Supplementary Figure 4. To know the interrelation between the metabolites, the metabolic closeness between different groups was analyzed using the correlation between the different metabolites (Supplementary Figure 5). Furthermore, DAMs were analyzed using the KEGG pathway database to find metabolic pathways that were considered the top 20 enriched (Supplementary Table 7). Interestingly, there were distinct differences in KEGG pathways across the four groups (A vs. B, B vs. C, C vs. D, and A vs. C). The most significantly enriched pathways were protein digestion and absorption; ABC transporters; biosynthesis of amino acids; aminoacyl-tRNA biosynthesis; mineral absorption; alanine, aspartate, and glutamate metabolism; glycine, serine, and threonine metabolism; arginine biosynthesis; and ovarian steroidogenesis. Among them, the pathways most closely related to vitamins ADE, Zn, and Se stress are mainly assigned to the digestive system, amino acid metabolism, translation, and endocrine system (Figures 7A–D). Relative levels of the differential metabolites of protein digestion and absorption (ko04974), alanine, aspartate, and glutamate metabolism (ko00250), aminoacyl-tRNA biosynthesis (ko00970), and aldosterone synthesis and secretion (ko04925) are shown *via* heatmap in all samples (Figures 7E–H). To further investigate the metabolites that changed the GCs development, we focused on the mineral absorption and ovarian steroidogenesis pathways in that the differential metabolites were demonstrated through the KEGG pathways mapper function and clustering heatmaps were drawn between different groups (Figure 8). In the four groups, the DAMs of mineral absorption consisted of D-glutamine, DL-serine, Glycine, Leucine, L-methionine, Phenylalanine, Proline, Alanine, Phosphoric acid, D- (+) -galactose, DL-threonine, and DL-tryptophan. Notably, in group C except for L-proline, DL-phenylalanine, alanine, and DL-threonine, the expression of the other metabolites was significantly higher than those of the other three groups. However, the expression of Alanine and DL-threonine was significantly higher in group D (Figure 8A). The DAMs of ovarian steroidogenesis consisted of arachidonic acid (peroxide free), 5-Androstene-3β,17β-diol, androstenedione, cholesterol, 17-alpha-hydroxyprogesterone, T, E_2_, and P_4_. Of note, in group C, except for arachidonic acid (peroxide free), the expression of other metabolites was significantly higher than those in the other three groups (Figure 8B). These data further suggested that vitamins ADE, Zn, and Se regulate the biological function of the GCs by affecting related metabolites.

**Table 1:** Evaluation parameters of the positive and negative ion model OPLS-DA model.

**NEG**							**POS**				
**Type**	**Group**	**A**	**N**	**R2X(cum)**	**R2Y(cum)**	**Q2(cum)**	**A**	**N**	**R2X(cum)**	**R2Y(cum)**	**Q2(cum)**
PCA	QC	5	28	0.529			4	28	0.532		
	A vs. B	3	12	0.598			2	12	0.535		
	B vs. C	3	12	0.543		-	3	12	0.578		
	C vs. D	3	12	0.565			2	12	0.521		
	A vs. C	3	12	0.519			3	12	0.592		
PLS-DA	A vs. B	3	12	0.521	0.999	0.939	2	12	0.522	1	0.981
	B vs. C	2	12	0.356	0.994	0.861	2	12	0.468	0.999	0.973
	C vs. D	2	12	0.359	0.988	0.826	2	12	0.512	0.999	0.98
	A vs. C	2	12	0.375	0.995	0.889	2	12	0.485	0.999	0.981
OPLS-DA	A vs. B	1 + 2	12	0.521	0.999	0.886	1 + 1	12	0.522	1	0.977
	B vs. C	1 + 1	12	0.356	0.994	0.84	1 + 1	12	0.468	0.999	0.965
	C vs. D	1 + 1	12	0.359	0.988	0.831	1 + 1	12	0.512	0.999	0.971
	A vs. C	1 + 1	12	0.375	0.995	0.83	1 + 1	12	0.485	0.999	0.971

PCA, principal component analysis; PLS-DA, partial least squares-discriminant analysis; OPLS-DA, orthogonal partial least squares discriminant analysis; QC, quality control; A: denotes the principal component score; R2X: denotes the model explanation rate; R2Y: indicates the explanation rate of the model for Y variable rate; Q2: indicates the predictive power of the model. In PLS-DA, OPLS-DA, Q2 is greater than 0.5, which indicates that the model is stable and reliable.

### Correlation analysis of the metabolome and transcriptome under vitamins ADE, Zn, and Se stress

**Figure 9:**
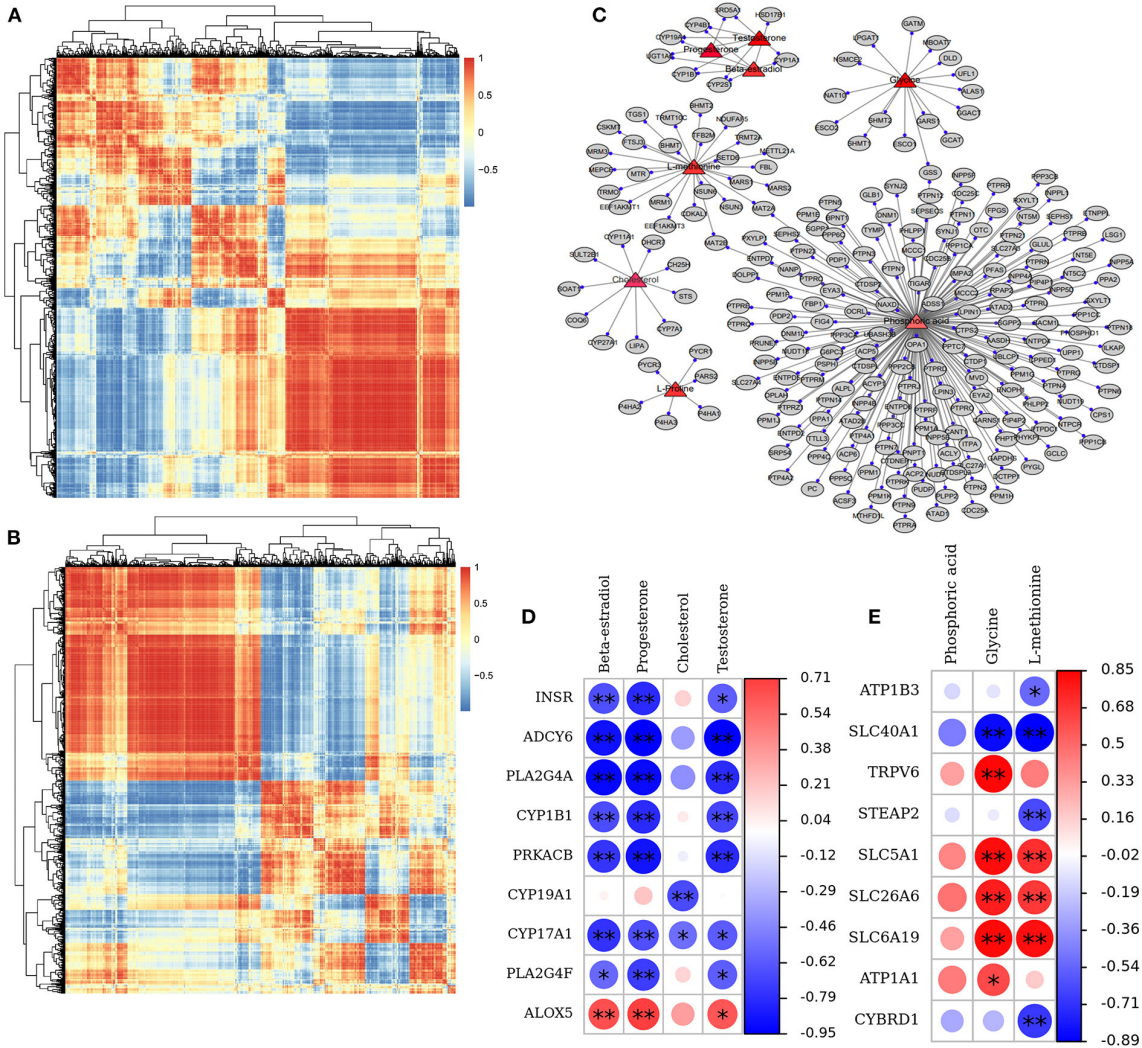
Correlation and pathway analysis of significant DAMs and DEGs. **(A,B)** Correlation analysis of the relationships between DEGs and DAMs. **(C)** The plot of targeting relationship network between metabolites and genes. **(D)** The correlation between nine DEGs and three key DAMs in the ovarian steroidogenesis pathway. **(E)** The correlation between nine DEGs and three key DAMs in the mineral absorption pathway. The *sign identifies the “gene-metabolite” with significant correlation; *p < 0.05, **p < 0.01.

To uncover the influence of the most important genes on the metabolic process in GCs of a donkey during the feeding of vitamins ADE, Zn, and Se mixture. We used correlation analysis targeting the relationships between differential metabolites and differential transcripts (Figures 9A,B). The expression levels of these genes were highly correlated with phosphoric acid, glycine, L-proline, L-methionine, E_2_, P_4_, cholesterol, and T identified in the GCs (Figure 9C). The ovarian steroidogenesis pathway involved in regulating GCs proliferation is highlighted. Nine DEGs (*INSR, CYP19A1, ALOX5, PLA2G4A, ADCY6, CYP1B1, PRKACB, CYP17A1*, and *PLA2G4F*) associated with the secretory function of GCs were annotated into the ovarian steroidogenesis, along with detected metabolic compounds: E_2_, cholesterol, P_4_ and T. Specifically, *INSR, ADCY6, CYP1B1, PRKACB, PLA2G4A, CYP17A1*, and *PLA2G4F* were a negative correlation with E_2_, P_4_, and T (*P* < 0.05); while *ALOX5* was a positive correlation with E_2_, P_4_, and T (*P* < 0.05); *CYP19A1* was a negative correlation with cholesterol (*P* < 0.01) (Figure 9D). Meanwhile, *STEAP2, CYBRD1, ATP1B3, ATP1A1, SLC26A6, SLC5A1, SLC6A19, SLC40A1*, and *TRPV6* were closely associated with the mineral absorption of GCs. Specifically, *SLC26A6, SLC5A1*, and *SLC6A19* were was a positive correlation with Glycine and L-methionine (*P* < 0.05). *SLC40A1* was a negative correlation with Glycine and L-methionine (*P* < 0.01). *TRPV6* and *ATP1A1* were positively associated with glycine (*P* < 0.05); while *ATP1B3* and *CYBRD1* were negatively related to L-methionine (*P* < 0.05; Figure 9E). These data demonstrated that these key genes interact with metabolites, suggesting that it may be important for the interchange of material and hormonal regulation between GCs and FF, which is required for GCs development.

## Discussion

Currently, vitamins ADE, Zn, and Se, as indispensable nutrients for ovarian function in feeding management practice, play an essential role in ovarian physiological functions. Studies have indicated that the level of fat-soluble vitamins is closely related to oocyte fertilization (vitamins A and E) and embryo development (vitamins A, E, and D) in FF (26). Other studies found that an adequate amount of Se and vitamin E supplementation may elevate ovarian reserve capacity and ensure homeostatic ovarian function (18). In addition, Zn is closely associated with the function of a multitude of enzymes and has an extremely critical role in the synthesis, secretion, and storage of compounds (27, 28). These studies above suggest that they play a very important role in the cross-talking among oocytes, GCs, and FF by affecting signaling, material transformation, and metabolism. In this study, we uncovered a previously unreported and pivotal function of vitamins ADE, Zn, and Se in accelerating follicular development and increasing ovulation rate and dominant follicle growth in jennies. Previous works have indicated that the follicle, as an important site of follicular development and ovulation, is a community of histological and functional structures, as well as important for the completion of a complex, precise, and communicative series of biological events between GCs and FF (29). Additionally, the fate of GCs is extremely closely related to follicular development and maturation, mainly through the precise regulation of a range of signaling and material exchange at the transcriptional, protein, and metabolic levels (30). Moreover, FF components may be altered directly or indirectly by hormone-mediated signaling *via* paracrine and autocrine signaling (31). Our results suggested that vitamins ADE, Zn, and Se may promote the proliferation and differentiation of GCs and follicular development by influencing key genes and metabolites mediated by mineral absorption and steroid hormones. It was reported that FF may influence oocyte quality, early embryo development, and subsequent pregnancy (32, 33). We found that there were molecular functions, signaling, and amino acid metabolites in FF. Furthermore, these key genes-metabolites may be important for signal transduction and substance metabolism in GCs, which is required for follicle development and maturation, but this specific molecular mechanism is not clear and needs to be further studied.

Vitamin A plays an important role in physiological processes, such as tricarboxylic acid cycle (TAC), steroid metabolism, and oocyte maturation (34). Meanwhile, vitamin D may improve polycystic ovary syndrome (PCOS), menstrual frequency, follicular development, and androgen (A) levels (35). While vitamin D deficiency is associated with hyperandrogenism in PCOS (36). A study found that biochemical markers for the diagnosis of hyperandrogenism, such as serum dehydroepiandrosterone, T, hormone-binding globulin, and free androgen index (37). Our results found that there was some level of T in follicular fluid and a negative correlation with genes of the steroid pathway, suggesting that vitamins are associated with metabolizing follicular reproduction. In addition, Parikh et al. reported that VDR is expressed in both mixed ovarian cells and a purified GCs culture, which is consistent with our results (38). Additionally, vitamin D induces 3-β-hydroxysteroid dehydrogenase expression and increases progesterone production and release (39). We also found that VDR was also expressed in GCs *via* the transcriptome expression profile, which suggested that vitamin D is involved in the reproductive processes. Ovarian steroidogenesis is regulated by hormonal signals, such as gonadotropin-releasing hormone (GnRH), follicle-stimulating hormone (FSH), and luteinizing hormone LH), and by several enzymes of which the majority belongs to the cytochrome P450 (CYP) superfamily (40). Correspondingly, our data revealed that C*YP2J2, CYP19A1, CYP1A1, CYP11A1, CYP17A1*, and *CYP1B1* are associated with E_2_, cholesterol, P_4_, and T in the ovarian steroidogenesis. Previous findings that *CYP1B1* carries out 4-hydroxylation of estradiol and exhibits minor metabolic activity toward 2-hydroxylation (41). More recently, metabolomics analysis revealed that α-tocopherol, a biologically active form of vitamin E, can be oxidized to several metabolites *via* β-oxidation and ω-oxidation by CYPs (42). Research indicated that genetic variations in *CYP1B1* and serum 25-hydroxyvitamin D levels on blood pressure (43). In addition, *CYP1B1* is involved in the metabolism of testosterone and progesterone, it is involved in the metabolism of vitamin A and shows a synergistic effect (44). Moreover, our data showed that CYPs might be an important regulator of GCs, further indicating that CYPs are required for follicle growth and maturation. Adenylyl cyclases (ADCY), by generating cyclic adenosine monophosphate (cAMP), play important roles in various cellular processes (45). We indicated that the levels of *ADCY4, ADCY8, ADCY3, ADCY1, ADCY6, ADCY9, ADCY5, ADCY7*, and *ADCY2* are significantly enriched in the ovarian steroidogenesis pathway. Similarly, ADCY catalyzes the production of cAMP from ATP, while phosphodiesterase degrades cAMP to 5-AMP in cell signaling, and the substance metabolism process is paramount (46). These results suggested that vitamins ADE, Zn, and Se may be involved in the steroid hormone signaling pathway by altering the secretion and metabolite content of ADCY, which in turn affects the proliferation and differentiation of GCs. Moreover, a recent study has also suggested that Zn is responsible for the function of over 300 enzymes, with a critical role in insulin synthesis, storage, secretion, and function (22, 47). It was involved in related biological processes by regulating the activity of protein tyrosine phosphatases in the IGF-1 signaling pathway (48). Our results are also similar to those of the above studies, suggesting that Zn may affect the levels of *IGF-1, STAR*, and *INSR* to participate in steroid hormone synthesis and secretion in GCs. In addition, it was reported that only the solute carrier (SLC) transporter family and the ATP-binding cassette family are present in a cell membrane transporter, and majority of small-molecule compounds functionally depend on SLC transporter (49). Our data provide direct evidence of mineral absorption that is dependent on the key genes of SLCs (*SLC11A2, SLC30A1, SLC31A1, SLC26A6, SLC5A1, SLC6A19, SLC40A1, SLC9A3*, and *SLC26A3*) and ATPs (*ATP1A2, ATP1B3, ATP1A1*, and *ATP1B1*). Furthermore, studies found that SLCs contribute to the cross-membrane transport of diverse substrates, including inorganic ions, amino acids, fatty acids, neurotransmitters (50), participating in many important physiological functions including nutrient supply, metabolic transformation, energy homeostasis, tissue development, oxidative stress, host defense, and neurological regulations (51). Our findings also showed these genes are closely associated with the metabolites (Alanine, D-(+)-galactose, DL-phenylalanine, DL-serine, DL-threonine, DL-tryptophan, L-proline, and proline) in the mineral absorption pathway. Especially, Glycine and L-methionine are associated with *TRPV6, ATP1A1, SLC26A6, SLC5A1*, and *SLC6A19*. These data suggested that the SLCs and ATPs were closely associated with the physiological functions and substance metabolism of GCs. In the future, the effect of adding single vitamins ADE, Zn, and Se to the development of GCs cultured *in vitro* will be investigated, investigating in depth the regulatory mechanisms between the screened key genes and metabolites further.

## Conclusion

In summary, our results suggest that exogenous supplementation with mixtures of vitamins ADE, Zn, and Se promote follicle development, preovulatory follicle maturation, and ovulation rate through the interaction of genes and metabolites of the ovarian steroidogenesis and mineral absorption, which are required for physiological function and metabolism of GCs. These outcomes may provide key insights into reproductive biology and fertility, exploring valuable biomarkers.

## Data availability statement

The data presented in the study are deposited in the National Center for Biotechnology Information Sequence Read Archive repository, accession number SRP387001.

## Ethics statement

The animal study was reviewed and approved by the Institutional Animal Care Committee of China Agricultural University.

## Author contributions

SZ, ZW, and YG designed the study. YG and WZ performed most of the experiment. YG wrote the manuscript. NL, SD, and HW contributed to the statistical analysis. SZ and ZW revised the manuscript. All authors read and approved the final manuscript.

## Funding

This research was funded by the Hebei Province project: The improvement project of the breeding innovation capacity of donkeys (Yang yuan) in 2022.

## Conflict of interest

The authors declare that the research was conducted in the absence of any commercial or financial relationships that could be construed as a potential conflict of interest.

## Publisher's note

All claims expressed in this article are solely those of the authors and do not necessarily represent those of their affiliated organizations, or those of the publisher, the editors and the reviewers. Any product that may be evaluated in this article, or claim that may be made by its manufacturer, is not guaranteed or endorsed by the publisher.
