# Supplementary material for: Integration analysis of metabolome and transcriptome reveals the effect of exogenous supplementation with mixtures of vitamins ADE, zinc, and selenium on follicular growth and granulosa cells molecular metabolism in donkeys (Equus asinus)
Source: Front Vet Sci. 2022 Oct 28;9:993426. doi: 10.3389/fvets.2022.993426 (PMC9650297; doi:10.3389/fvets.2022.993426)
Supplement: Supplementary file 1 [file Data_Sheet_1.pdf]

# Supplementary Material

**For**

**Integration Analysis of Metabolome and Transcriptome Reveals the Effect of Exogenous Supplementation with Mixtures of Vitamins ADE, Zn, and Se on Follicular Growth and Granulosa Cells Molecular Metabolism in Donkeys (*Equus asinus*)**

Yajun Guo[1], Weisen Zhao[1], Nan Li[2], ShiZhen Dai[1], Hao Wu[1], Zhenlong Wu[3], Shenming Zeng[1]*

[1]*National Engineering Laboratory for Animal Breeding, Key Laboratory of Animal Genetics and Breeding of the Ministry of Agriculture, College of Animal Science and Technology, China Agricultural University, Beijing, China*

[2] *Department of Clinical Sciences, College of Veterinary Medicine, China Agricultural University, Beijing, China*

[3]*State Key Laboratory of Animal Nutrition, College of Animal Science and Technology, China Agricultural University, Beijing, China*

*Corresponding author: Shenming Zeng, Email: zengsm@cau.edu.cn

# Supplementary Information Text

## Materials and Methods

### Ultrasound scanning for follicular diameter

The experiments were performed according to the method of ultrasound examination of donkey follicle morphology and the specific protocols for determining follicle development and ovulation in the references (1, 2). To be specific, the jennies must be adequately restrained before examination of the ovarian follicles. They should be placed in stocks before the examination. A water-based lubricant such as methylcellulose can be used. The lubricated hand should then be shaped into a cone and gently inserted into the rectum through the tight anal sphincter. During the examination, the more frequently used hand can be used to perform the ultrasonic scanner probe with a 6.5 MHz linear array probe (KX5600, Kaixin Electronic Instrument Co., Ltd, Xuzhou, China) by the other hand operates the ultrasound machine. Slow rotation of the arm in conjunction with gentle forward pressure enhances advancement into the rectum. The leading edge of the uterus can be detected by first inserting the arm deeply into the rectum, cupping the hand downward, then slowly retracting the arm. The ovary can usually be located with the fingertips or slowly rotate the probe with your arm and find the location of the ovaries. The ultrasound probe was fixed, the ultrasound instrument was operated, the follicle diameter was measured, and the images were saved. Several ultrasound images of different follicle diameters are shown in **Supplementary Figure 1B-C.**

### Using a vaginal vault ultrasound puncture device for follicular fluid

The protocol for transvaginal ultrasound-guided follicle aspiration and the method for determining follicle size was collected with reference to that described previously (3, 4, 5,

6). To ensure consistency in follicle size, the probe attached is used a vagina to visualize ovarian follicles for transvaginal aspiration. The veterinary ultrasound machine's measuring imaging system ((Easi-Scan Micro-Convex, BCF Technology Ltd, Scotland, UK; Probe parameter: 15 mm radius, frequency range 5 MHz to 8 MHz, 80 element crystal array, 10 digital channels, 90º curved) guided the aspiration of follicular fluid from the follicles that could be satisfied with (**Supplementary Figure1D**) follicles (Diameter: 30-40mm) were collected from each donkey during three natural estrous cycles (collection of 2 follicles in an estrous cycle). To be specific, transvaginal aspiration is performed with a transvaginal probe handle into which the ultrasound probe is mounted. The probe handles had within it a channel for the needle. In the tranquilized jenny, the probe is placed in the vagina, and the ovary is manipulated via palpation per rectum. A specialized needle, typically a 12- to 17-gauge double-lumen needle, is placed through the guide channel of the probe handle. The ovary is imaged through the vaginal wall and manipulated by the hand per rectum so that the follicle is placed in the path of the needle as visualized on the ultrasound screen. The needle is then guided forward through the vaginal wall into the follicle, and the contents of the follicle are aspirated, typically with a vacuum pump.

## References


1. Li N, Yang F, Yu J, Yang W, Wu S, Ma J, et al. Characteristics of follicular dynamics and reproductive hormone profiles during oestrous cycles of jennies over an entire year. *Reprod Domest Anim.* (2021), 56:448-458.
2. Yang F, Li N, Liu B, Yu J, Wu S, Zhang R, et al. Practical protocols for timed artificial insemination of jennies using cooled or frozen donkey semen. *Equine Vet J.* (2021), 53:1218–1226.
3. Carnevale EM, Coutinho da Silva MA, Panzani D, Stokes JE, Squires EL. Factors affecting the success of oocyte transfer in a clinical program for subfertile mares. *Theriogenology.* (2005), 64:519–527.
4. Hinrichs K. Assisted reproductive techniques in mares. *Reprod Domest Anim.* (2018),53 Suppl 2:4-13.
5. Manjunatha BM, Gupta PS, Ravindra JP, Devaraj M, Nandi S. In vitro embryo development and blastocyst hatching rates following vitrification of river buffalo embryos produced from oocytes recovered from slaughterhouse ovaries or live animals by ovum pick-up. *Anim Reprod Sci.* (2008),104:419-426.
6. Galli C, Crotti G, Notari C, Turini P, Duchi R, Lazzari G. Embryo production by ovum pick up from live donors. *Theriogenology.* (2001),55:1341-1357.

# Supplementary Figures

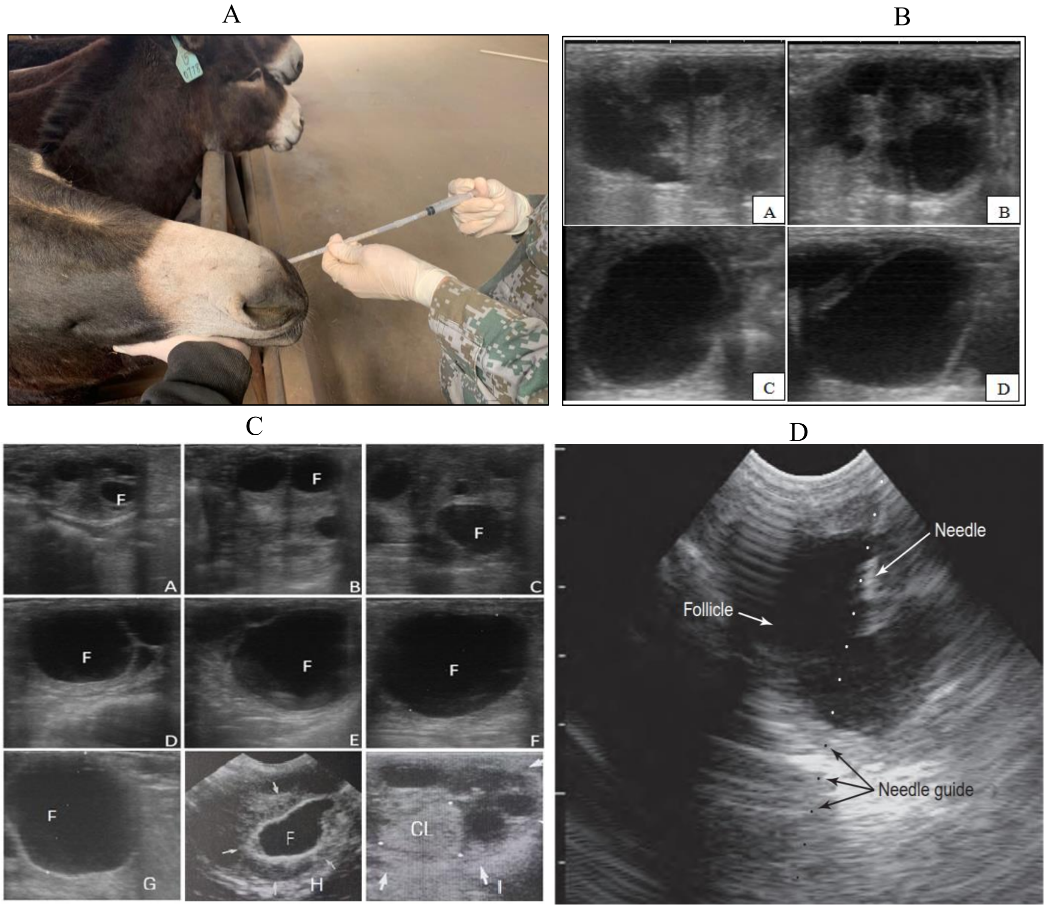


**Supplementary Figure 1**. Feeding experiment live image and B-ultrasound scanner testing ovarian follicles in donkey factory. **(A)** The mixture (vitamin ADE, Zn, and Se) was infused through a syringe without a needle and completely swallowed in the mouth of the donkey until absorption and digestion, once a day at 5 mL per jenny continuously for 2 months. **(B)** B-ultrasound scanner was used to observe one estrus cycle continuously (21 days) to examine follicular development. A: Small follicles ($\Phi < 20$ mm). B: Growth follicles (20 mm $< \Phi <$ 25 mm). C: Antral follicles (30 mm $< \Phi <$ 40mm). D: Pre-ovulation follicles ($\Phi >$ 40mm). **(C)** The morphological changes during follicular development are shown. The follicles in Figures A to F show the continuous development and enlargement of the follicle (10mm-20mm-25mm-30mm-35mm-40mm in diameter, respectively). G shows a marked thickening of the follicular rim indicating imminent ovulation, H shows a collapsed and deformed follicle in the process of ovulation, and the image in I shows the formation of the corpus luteum after ovulation. **(D)**: Ovarian follicle punctured during transvaginal aspiration procedure. The etched tip of the special needle is echogenic, allowing visual confirmation that the tip is within the follicle antrum.

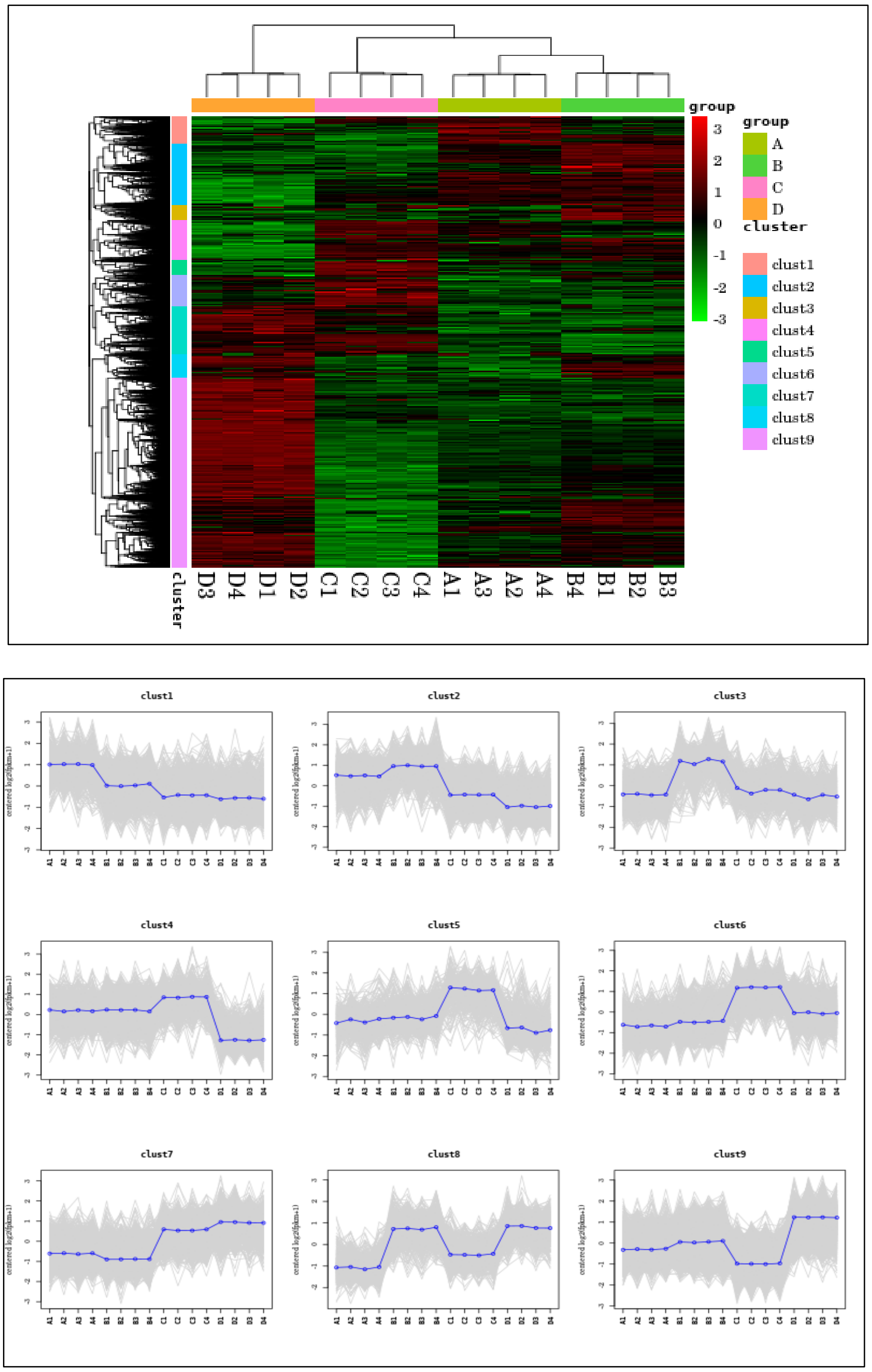


**Supplementary Figure 2**. Cluster analysis of expression levels in different samples. Horizontal representation of genes, one sample per column, red for high expression genes, green for low expression genes, A1-A4: 50% group; B1-B4: 75% group; C1-C4: 100% group; D1-D4: 150% group.

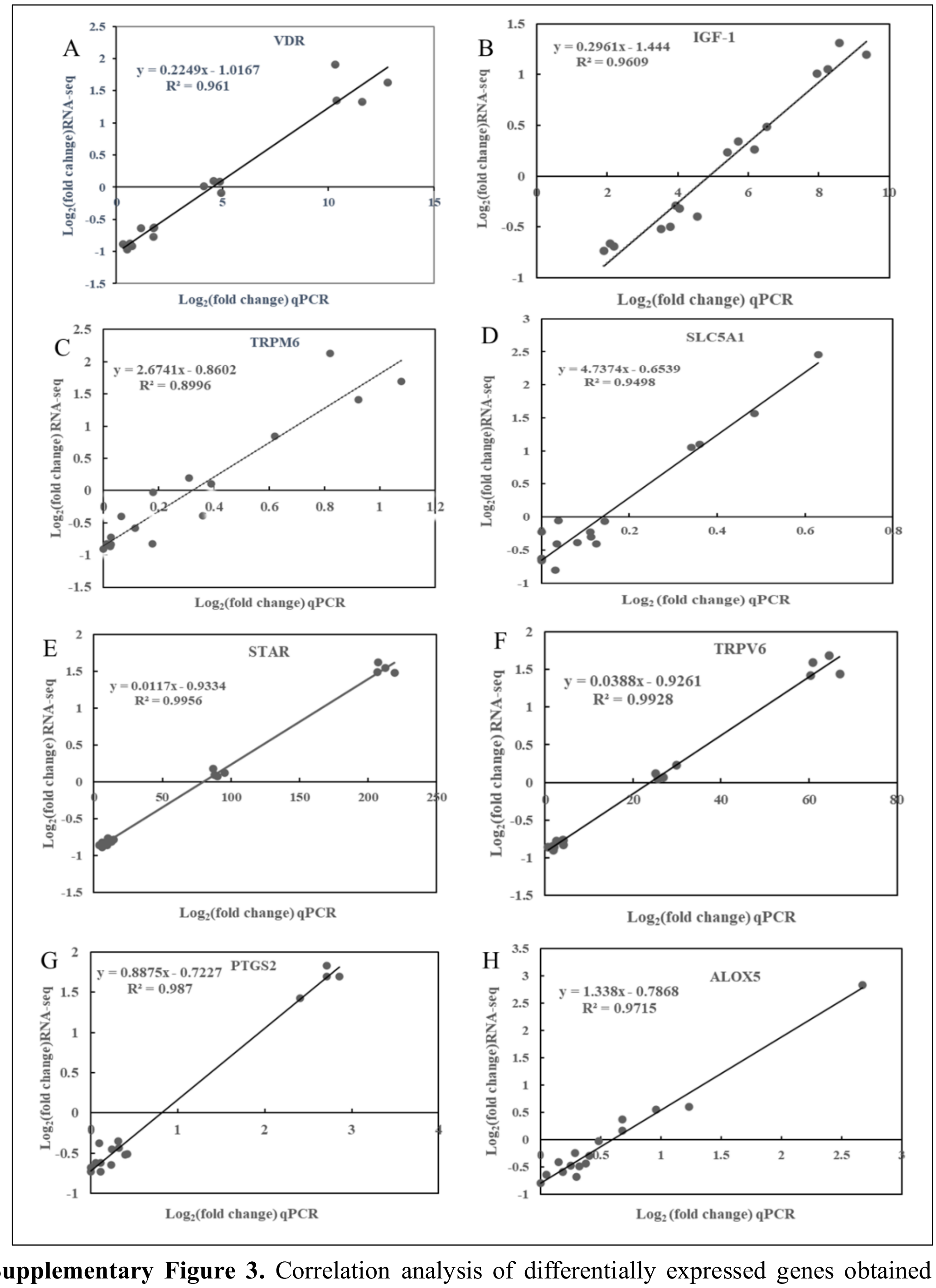


**Supplementary Figure 3.** Correlation analysis of differentially expressed genes obtained from RNA-seq and qPCR. **(A-H)** The qPCR values were normalized relative to the expression levels of GADPH in the same sample. Data are expressed as the mean of four biological replicates.

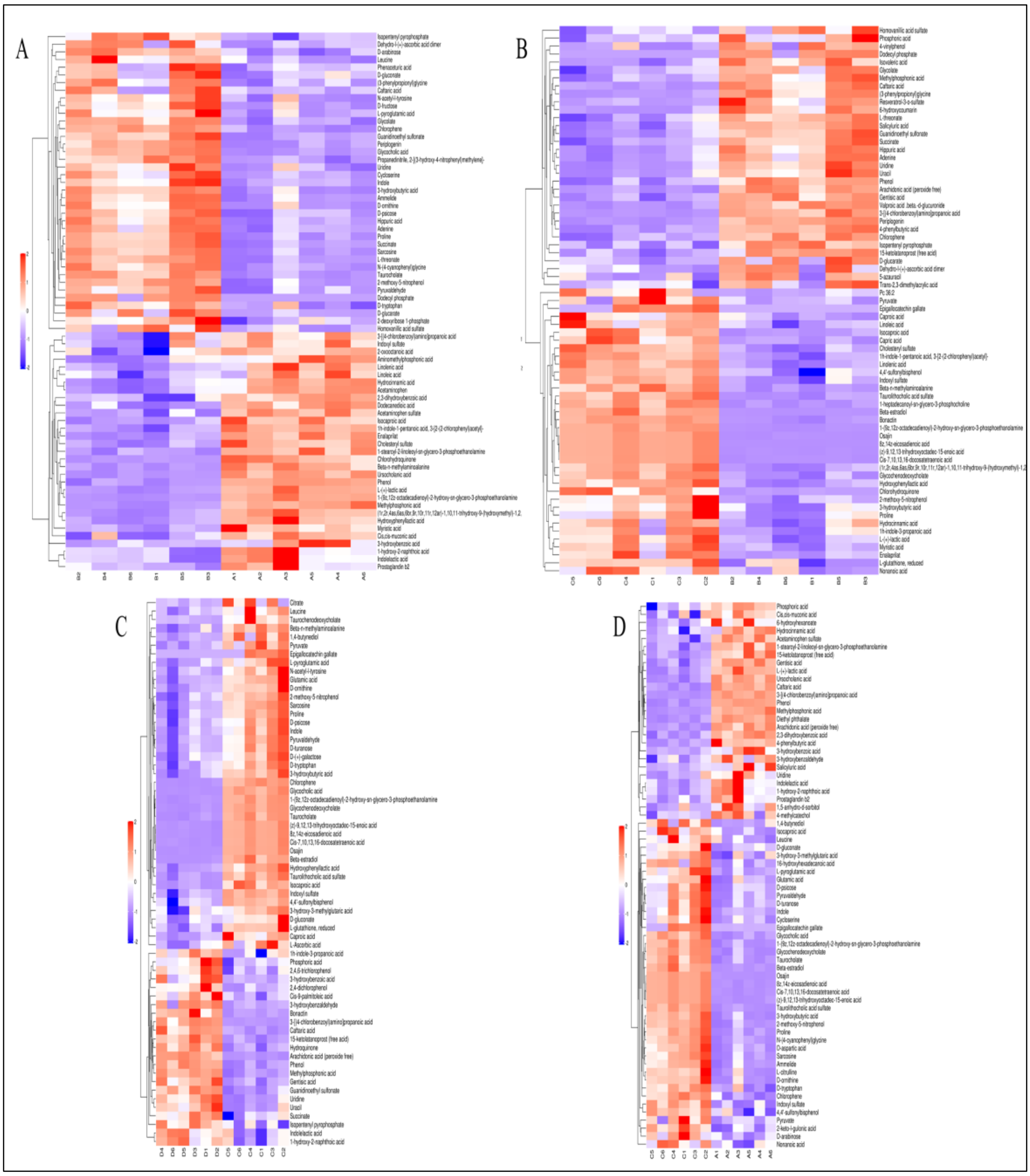


**Supplementary Figure 4.** Overview of significant difference metabolite hierarchical clustering heat map. **(A-D)** The total metabolite heat map derived from LC-MS/MS (non-targeted metabolome) profiling. **(A)** Difference metabolite hierarchical clustering heat map in A VS. B; **(B)** Difference metabolite hierarchical clustering heat map in B VS. C; **(C)** Difference metabolite hierarchical clustering heat map in C VS. D; **(D)** Difference metabolite hierarchical clustering heat map in A VS. C. A: 50% group; B: 75% group; C: 100% group; D: 150% group. Metabolomics usually uses strict OPLS-DA VIP > 1 and $P < 0.05$ as screening criteria for significantly different metabolites.

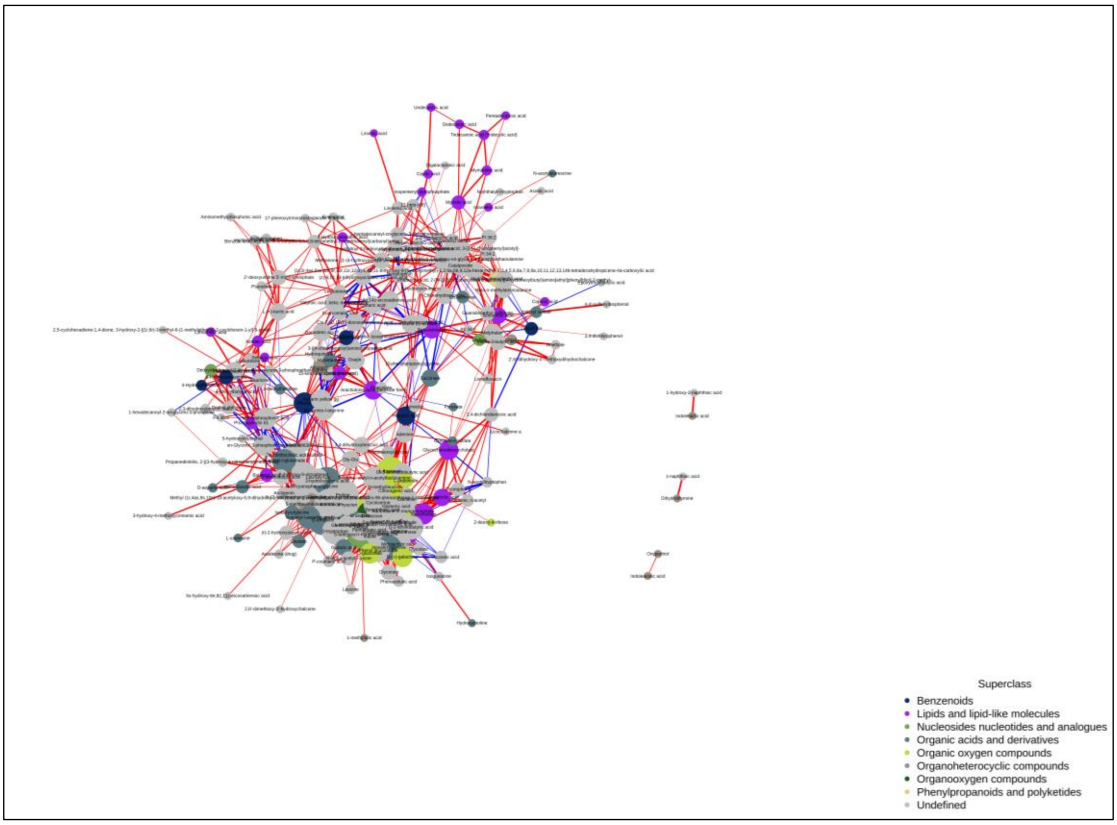


**Supplementary Figure 5:** Correlation analysis of the inter-regulatory relationships between metabolites. The dots in the graph represent significantly different metabolites, and the size of the dots correlates with the degree of connectivity, the larger the degree, the larger the dots. The color of the line represents the correlation, red indicates a positive correlation, and blue indicates a negative correlation. The thickness of the line represents the absolute correlation coefficient. The thicker the line, the larger the correlation. Correlation coefficient $|r| > 0.8$ and $P < 0.05$ metabolite molecules.

## Supplementary Tables

**Supplementary Table 1. Basic diet formula for adult female donkeys**

| Categories | Content（g/100Kg Weight） |
|---|---|
| Millet straw | 4650 |
| Soybean meal | 150 |
| Corn flour | 435 |
| Mountain flour | 37.5 |
| Calcium hydrophosphate | 7.5 |
| Salt | 37.5 |
| Base premix | 7.5 |

**Supplementary Table 2. Recipe of Vitamin ADE, Zn and Se mixture**

| Groups | VA (IU/100Kg Weight) | VD (IU/100Kg Weight) | VE (IU/100Kg Weight) | Se (mg/100Kg Weight) | Zn (mg/100Kg Weight） |
|---|---|---|---|---|---|
| A (50% mixture) | 7500 | 825 | 125 | 0.1875 | 75 |
| B (75% mixture) | 10500 | 1155 | 175 | 0.2625 | 105 |
| C (100% mixture) | 15000 | 1650 | 250 | 0.375 | 150 |
| D (150% mixture) | 22500 | 2475 | 375 | 0.5625 | 225 |

Note: The mixture contents of group C are derived from the equine NRC standard (2007), whereas groups A, B and D contain 0.5, 0.75 and 1.5 times more mixture than group C-50%, 75%, and 150%, respectively. the mixture: vitamin ADE, Zn, and Se.

**Supplementary Table3. List of information primer sequences in qPCR**

| Genes | Sequences (5′-3′)<br>F: Forward; R: Reverse | Accession No. | Length/bp |
|---|---|---|---|
| *GAPDH* | F: GTAAACGGATTTGGCCGTATTG<br>R: GCCATGGGTGGAATCATACT | XM_044756267 | 141 |
| *VDR* | F: TGTTCACCTGTCCCTTCAAC<br>R: TCCTTCATCATGCCGATGTC | XM_044755916.1 | 106 |
| *TRPM6* | F: GTGACATCAGAGAGGGTTACAG<br>R: CACCTGGCTGTCCAAAGATA | XM_044767632.1 | 102 |
| *TRPV6* | F: GGTGGAAGACAAACAGGATCTC<br>R: CGATTCCTTGTCCACCTCATC | XM_014845207.2 | 97 |
| *SLC5A1* | F: ACCTGGCCATCATTCTCTTATT<br>R: AAGCAAACCCAGTCAGGATAG | XM_014834194.1 | 135 |
| *ALOX5* | F: GGTGAACTTTGGCCAGTATGA<br>R: GATCTGCTCGATGGTGACTATG | XM_044744143.1 | 106 |
| *STAR* | F: AGCATGGTCCCACTTGTATG<br>R: TGAAAGGACCTGGTTGATGATAG | XM_014831108.1 | 128 |
| *PTGS2* | F: TGCCTCAATTCAGTCTCTCATC<br>R: GTGGGATTCACGTCATCTAGTC | XM_014865790.1. | 141 |
| *IGF-1* | F: CTGAGGAGGCTGGAGATGTA<br>R: CTTCTGAGCCTTGGGCATATC | XM_014835027.1 | 99 |

**Supplementary Table 4. Effects of different doses of vitamin ADE, Zn, and, Se on ovulation rate**

| Groups | Single dominant follicle rate (%) | Bilateral dominant follicle rate (%) | Single ovulation rate (%) | Bilateral ovulation rate (%) |
|---|---|---|---|---|
| 150% | 68.42(13/19) | 31.58 $^{b}$ (6/19) | 78.95 (15/19) | 21.05 $^{b}$ (4/19) |
| 100% | 58.33(14/24) | 33.33 $^{b}$ (8/24) | 62.50 (15/24) | 37.50 $^{a}$ (9/24) |
| 75% | 35.71(5/14) | 64.29 $^{a}$ (9/14) | 64.29 (9/14） | 35.71 $^{a}$ (5/14) |
| 50% | 85.71(12/14) | 14.29 $^{c}$ (2/14) | 85.71 (12/14) | 14.29 $^{c}$ (2/14) |

Note： The values with different superscripts in the same row significantly differ, $P < 0.05$.

**Supplementary Table 5-1. Raw data processing and quality control**

| Samples | Raw_ Read_ Number | Raw_ Bases | Raw_Q30_ number | Raw_N (%) | Raw_Q20 (%) | Raw_ Q30 (%) |
|---|---|---|---|---|---|---|
| A1 | 44924056 | 6738608400 | 6375556752 | 0.000174 | 98.03 | 94.61 |
| A2 | 39080490 | 5862073500 | 5546915352 | 0.000175 | 98.03 | 94.62 |
| A3 | 45084682 | 6762702300 | 6386368354 | 0.000171 | 97.95 | 94.43 |
| A4 | 42890134 | 6433520100 | 6105194243 | 0.000172 | 98.15 | 94.89 |
| B1 | 41816468 | 6272470200 | 5832174631 | 0.000136 | 97.24 | 92.98 |
| B2 | 41974248 | 6296137200 | 5851980714 | 0.000141 | 97.20 | 92.94 |
| B3 | 46364990 | 6954748500 | 6461678318 | 0.000139 | 97.21 | 92.91 |
| B4 | 42102362 | 6315354300 | 5856068768 | 0.000137 | 97.14 | 92.72 |
| C1 | 36150398 | 5422559700 | 5060884987 | 0.000139 | 97.38 | 93.33 |
| C2 | 40797686 | 6119652900 | 5691822163 | 0.00014 | 97.22 | 93.00 |
| C3 | 43061416 | 6459212400 | 5980824259 | 0.000507 | 97.03 | 92.59 |
| C4 | 45320334 | 6798050100 | 6327659389 | 0.000466 | 97.27 | 93.08 |
| D1 | 39295292 | 5894293800 | 5497983157 | 0.000137 | 97.43 | 93.27 |
| D2 | 41770010 | 6265501500 | 5830574254 | 0.000141 | 97.34 | 93.05 |
| D3 | 42368068 | 6355210200 | 5905764965 | 0.000475 | 97.27 | 92.92 |
| D4 | 38220694 | 5733104100 | 5332061571 | 0.00014 | 97.30 | 93.00 |

Sample: Sample Reads No.: Total number of Reads; bases(bp): Total number of bases; Q30 (bp): Total number of bases with 99.9% or higher base identification accuracy; N (%): Percentage of ambiguous bases; Q20(%): Percentage of bases with base identification accuracy above 99%; Q30(%): Percentage of bases with base identification accuracy above 99.9%.

**Supplementary Table 5-2. Alignment analysis with the reference genome**

| Samples | Clean_Reads | Total_Mapped | Multiple_Mapped | Uniquely_Mapped | Map_Events | Mapped_to_Gene | Mapped_to_InterGene | Mapped_to_Exon |
|---|---|---|---|---|---|---|---|---|
| A1 | 41079858 | 37753739 (91.90%) | 1145254 (3.03%) | 36608485 (96.97%) | 36608485 | 26449450 (72.25%) | 10159035 (27.75%) | 23127609 (87.44%) |
| A2 | 36066250 | 33130556 (91.86%) | 981625 (2.96%) | 32148931 (97.04%) | 32148931 | 23168210 (72.07%) | 8980721 (27.93%) | 20257307 (87.44%) |
| A3 | 41497718 | 38110445 (91.84%) | 1160279 (3.04%) | 36950166 (96.96%) | 36950166 | 26725193 (72.33%) | 10224973 (27.67%) | 23387833 (87.51%) |
| A4 | 39593424 | 36398999 (91.93%) | 1074100 (2.95%) | 35324899 (97.05%) | 35324899 | 25396996 (71.90%) | 9927903 (28.10%) | 22204497 (87.43%) |
| B1 | 38565806 | 32594840 (84.52%) | 927621 (2.85%) | 31667219 (97.15%) | 31667219 | 22256065 (70.28%) | 9411154 (29.72%) | 18910608 (84.97%) |
| B2 | 38746954 | 32511552 (83.91%) | 920959 (2.83%) | 31590593 (97.17%) | 31590593 | 22193395 (70.25%) | 9397198 (29.75%) | 18861895 (84.99%) |
| B3 | 42640498 | 35768502 (83.88%) | 1006232 (2.81%) | 34762270 (97.19%) | 34762270 | 24322157 (69.97%) | 10440113 (30.03%) | 20598521 (84.69%) |
| B4 | 38873972 | 32920491 (84.69%) | 926116 (2.81%) | 31994375 (97.19%) | 31994375 | 22373041 (69.93%) | 9621334 (30.07%) | 18912257 (84.53%) |
| C1 | 33125638 | 29984207 (90.52%) | 948758 (3.16%) | 29035449 (96.84%) | 29035449 | 20749974 (71.46%) | 8285475 (28.54%) | 18759333 (90.41%) |
| C2 | 37464314 | 33901497 (90.49%) | 1086423 (3.20%) | 32815074 (96.80%) | 32815074 | 23599902 (71.92%) | 9215172 (28.08%) | 21360823 (90.51%) |
| C3 | 39700020 | 35888412 (90.40%) | 1174302 (3.27%) | 34714110 (96.73%) | 34714110 | 24931615 (71.82%) | 9782495 (28.18%) | 22561994 (90.50%) |
| C4 | 41808378 | 37943782 (90.76%) | 1229300 (3.24%) | 36714482 (96.76%) | 36714482 | 26228750 (71.44%) | 10485732 (28.56%) | 23697296 (90.35%) |
| D1 | 36206326 | 32992772 (91.12%) | 865916 (2.62%) | 32126856 (97.38%) | 32126856 | 22781796 (70.91%) | 9345060 (29.09%) | 18161194 (79.72%) |
| D2 | 38571372 | 35086180 (90.96%) | 906598 (2.58%) | 34179582 (97.42%) | 34179582 | 24265048 (70.99%) | 9914534 (29.01%) | 19370706 (79.83%) |
| D3 | 38971726 | 35486364 (91.06%) | 933362 (2.63%) | 34553002 (97.37%) | 34553002 | 24534366 (71.01%) | 10018636 (28.99%) | 19534995 (79.62%) |
| D4 | 35309320 | 32081642 (90.86%) | 848935 (2.65%) | 31232707 (97.35%) | 31232707 | 22227792 (71.17%) | 9004915 (28.83%) | 17739955 (79.81%) |

**Supplementary Table 6. Differential metabolites in A and C groups (VIP >7).**

| Name | VIP | Fold change(C/A) | p-value | m/z | rt(s) | SuperClass |
|---|---|---|---|---|---|---|
| **Pyrantel** | 40.500 | 0.351 | 0.00000 | 207.095 | 65.293 | Organoheterocyclic compounds |
| **Lpc 18:2** | 30.351 | 0.275 | 0.00000 | 520.340 | 280.042 | Lipids and lipid-like molecules |
| **Cis,cis-muconic acid** | 23.257 | 0.793 | 0.04946 | 141.016 | 437.242 | Lipids and lipid-like molecules |
| **L-(+)-lactic acid** | 19.487 | 0.668 | 0.00001 | 89.023 | 327.928 | Organic acids and derivatives |
| **Arginine** | 18.766 | 1.291 | 0.00014 | 175.119 | 504.192 | Organic acids and derivatives |
| **L-carnitine** | 18.552 | 1.297 | 0.00000 | 162.113 | 378.399 | Organic nitrogen compounds |
| **1-palmitoyl-sn-glycero-3-phosphocholine** | 15.801 | 0.665 | 0.00000 | 496.340 | 280.656 | Lipids and lipid-like molecules |
| **1-Stearoyl-sn-glycerol 3-phosphocholine (LPC (18:0))** | 15.231 | 0.752 | 0.00013 | 524.371 | 277.690 | Lipids and lipid-like molecules |
| **Ddao** | 14.479 | 0.041 | 0.00000 | 202.217 | 269.281 | Organic nitrogen compounds |
| **Bergaptol** | 14.279 | 1.151 | 0.00442 | 203.053 | 361.571 | Phenylpropanoids and polyketides |
| **N-octadecylamine** | 14.004 | 0.383 | 0.00000 | 270.315 | 277.495 | Organic nitrogen compounds |
| **1-palmitoyl-2-linoleoyl-sn-glycero-3-phosphocholine** | 13.774 | 0.713 | 0.01176 | 758.571 | 253.838 | Lipids and lipid-like molecules |
| **Ng,ng-dimethyl-l-arginine** | 13.514 | 1.642 | 0.00000 | 203.150 | 479.514 | Organic acids and derivatives |
| **1-oleoyl-sn-glycero-3-phosphocholine** | 13.400 | 0.524 | 0.00000 | 522.356 | 278.479 | Lipids and lipid-like molecules |

| | | | | | | |
|---|---|---|---|---|---|---|
| **Lobelanidine** | 13.085 | 0.018 | 0.00000 | 202.180 | 263.860 | Organic nitrogen compounds |
| **Pyruvate** | 11.543 | 1.300 | 0.03019 | 87.008 | 226.304 | Organic acids and derivatives |
| **DL-arginine** | 9.849 | 1.384 | 0.00000 | 116.071 | 362.973 | Organic acids and derivatives |
| **D-glutamine** | 9.846 | 1.458 | 0.00003 | 147.077 | 384.981 | Organic acids and derivatives |
| **Creatine** | 9.506 | 1.230 | 0.00013 | 132.077 | 376.945 | Organic acids and derivatives |
| **Glycerophosphocholine** | 9.483 | 1.163 | 0.00134 | 104.107 | 353.245 | Lipids and lipid-like molecules |
| **Urea** | 9.346 | 1.170 | 0.00064 | 61.040 | 245.410 | Organic acids and derivatives |
| **Methylphosphonic acid** | 8.487 | 0.181 | 0.00000 | 94.980 | 221.478 | Organic acids and derivatives |
| **Stachydrine** | 8.394 | 1.445 | 0.00000 | 144.102 | 344.856 | Organic acids and derivatives |
| **1-methyl-l-histidine** | 8.331 | 3.272 | 0.00544 | 170.092 | 395.891 | Organic acids and derivatives |

**Supplementary Table 7. The top 20 KEGG pathways of differential metabolites**

| Map_ Name | Test | Test All | Ref | Ref All | Test_ per | Ref_per | p. value | FDR | Rich Factor |
|---|---|---|---|---|---|---|---|---|---|
| Protein digestion and absorption | 21.000 | 381.000 | 47.000 | 5900.000 | 5.512 | 0.797 | 0.000000 | 0.000000 | 0.447 |
| ABC transporters | 33.000 | 381.000 | 137.000 | 5900.000 | 8.661 | 2.322 | 0.000000 | 0.000000 | 0.241 |
| Biosynthesis of amino acids | 27.000 | 381.000 | 128.000 | 5900.000 | 7.087 | 2.169 | 0.000000 | 0.000002 | 0.211 |
| Aminoacyl-tRNA biosynthesis | 16.000 | 381.000 | 52.000 | 5900.000 | 4.199 | 0.881 | 0.000000 | 0.000003 | 0.308 |
| Mineral absorption | 12.000 | 381.000 | 29.000 | 5900.000 | 3.150 | 0.492 | 0.000000 | 0.000003 | 0.414 |
| Alanine, aspartate and glutamate metabolism | 11.000 | 381.000 | 28.000 | 5900.000 | 2.887 | 0.475 | 0.000001 | 0.000019 | 0.393 |
| Glycine, serine and threonine metabolism | 14.000 | 381.000 | 50.000 | 5900.000 | 3.675 | 0.847 | 0.000002 | 0.000056 | 0.280 |
| Arginine biosynthesis | 9.000 | 381.000 | 23.000 | 5900.000 | 2.362 | 0.390 | 0.000006 | 0.000165 | 0.391 |
| Ovarian steroidogenesis | 8.000 | 381.000 | 24.000 | 5900.000 | 2.100 | 0.407 | 0.000083 | 0.001763 | 0.333 |
| Neuroactive ligand-receptor interaction | 12.000 | 381.000 | 52.000 | 5900.000 | 3.150 | 0.881 | 0.000086 | 0.001763 | 0.231 |
| Arginine and proline metabolism | 15.000 | 381.000 | 78.000 | 5900.000 | 3.937 | 1.322 | 0.000110 | 0.002047 | 0.192 |
| Cholesterol metabolism | 5.000 | 381.000 | 10.000 | 5900.000 | 1.312 | 0.169 | 0.000210 | 0.003592 | 0.500 |
| Prolactin signaling pathway | 5.000 | 381.000 | 11.000 | 5900.000 | 1.312 | 0.186 | 0.000365 | 0.005612 | 0.455 |
| Biosynthesis of various secondary metabolites - part 3 | 11.000 | 381.000 | 52.000 | 5900.000 | 2.887 | 0.881 | 0.000383 | 0.005612 | 0.212 |
| Synaptic vesicle cycle | 5.000 | 381.000 | 12.000 | 5900.000 | 1.312 | 0.203 | 0.000593 | 0.008105 | 0.417 |
| cAMP signaling pathway | 7.000 | 381.000 | 25.000 | 5900.000 | 1.837 | 0.424 | 0.000770 | 0.009866 | 0.280 |
| Bile secretion | 15.000 | 381.000 | 97.000 | 5900.000 | 3.937 | 1.644 | 0.001260 | 0.015192 | 0.155 |
| Phenylalanine metabolism | 11.000 | 381.000 | 60.000 | 5900.000 | 2.887 | 1.017 | 0.001364 | 0.015530 | 0.183 |
| GABAergic synapse | 4.000 | 381.000 | 9.000 | 5900.000 | 1.050 | 0.153 | 0.001662 | 0.017936 | 0.444 |
| Cysteine and methionine metabolism | 11.000 | 381.000 | 63.000 | 5900.000 | 2.887 | 1.068 | 0.002058 | 0.019962 | 0.175 |
